# The Purification, Characterization, and Biological Activity of New Polyketides from Mangrove-Derived Endophytic Fungus *Epicoccum nigrum* SCNU-F0002

**DOI:** 10.3390/md17070414

**Published:** 2019-07-12

**Authors:** Zhangyuan Yan, Shitong Wen, Meng Ding, Huixian Guo, Cuiying Huang, Xintong Zhu, Junyi Huang, Zhigang She, Yuhua Long

**Affiliations:** 1 School of Chemistry and Environment, South China Normal University, Guangzhou 510006, China; 2 School of Chemistry, Sun Yat-Sen University, Guangzhou 510275, China

**Keywords:** benzofuranone derivative, coumarin, dihydroradicinin, antioxidant activity, antibacterial effect

## Abstract

Six new polyketides, including one coumarin (**1**), two isocoumarins (**2** and **3**), dihydroradicinin (**4**), and two benzofuranone derivatives (**7** and **8**), together with seven known analogues (**5**–**6** and **9**–**13**) were isolated from the culture of the mangrove endophytic fungus *Epicoccum nigrum* SCNU-F0002. The structures were elucidated on the interpretation of spectroscopic data. The absolute configuration of Compounds **2** and **3** were determined by comparison of their ECD spectra with the data of their analogue dihydroisocoumarins described in the literature. The absolute configuration of **4** was determined by single-crystal *X*-ray diffraction. All the compounds were screened for their antioxidant, antibacterial, anti-phytopathogenic fungi and cytotoxic activities. Using a DPPH radical-scavenging assay, Compounds **10**–**13** showed potent antioxidant activity with IC_50_ values of 13.6, 12.1, 18.1, and 11.7 μg/mL, respectively. In addition, Compounds **6** and **7** showed antibacterial effects against *Bacillus subtilis* (ATCC 6538)*, Escherichia coli* (ATCC 8739), and *Staphylococcus aureus* (ATCC 6538), with MIC values in the range of 25–50 μg/mL.

## 1. Introduction 

**Figure 1:**
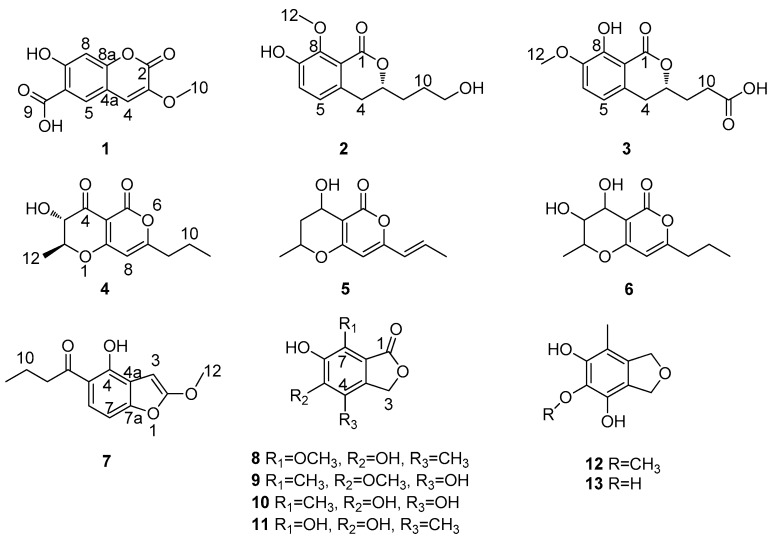
Chemical structures of **1**–**13**.

Marine natural products play a significant role in the drug discovery and development process [1]. Marine-derived fungi are widely recognized as an emerging source for the production of novel and bioactive secondary metabolites [2]. Marine fungi associated with mangroves live in extreme ecosystems characterized by high salinity, which may determine the production of unique metabolites [3,4]. Exploring the secondary metabolites with excellent biological activity and pharmacy value from mangrove-derived fungi has attracted great attention of both pharmaceutical and natural product chemists [1,2,3,4]. Different species belonging to the genus *Epicoccum* have been reported to produce many bioactive secondary metabolites with antiviral [5], antibacterial [6], antifungal [7], anti-inflammatory [8], and cytotoxic activities [9]. As part of our ongoing research on bioactive compounds from mangrove endophytic fungi [10,11], the chemical investigation of the ethyl acetate extract of the endophytic fungus *Epicoccum nigrum* SCNU-F0002, which was isolated from the fresh fruit of the mangrove plant *Acanthus ilicifolius* L., yielded five new polyketide compounds (**1**, **2**, **3**, **7**, and **8**) and one fungus-derived polyketide (**4**), which was previously semi-synthesized from radicinin [12], together with seven known compounds (**5**,**6**, and **9**–**13**) (Figure 1). In this paper, we report the purification, structure elucidation, and bioactivities of these compounds.

## 2. Results

**Figure 2:**
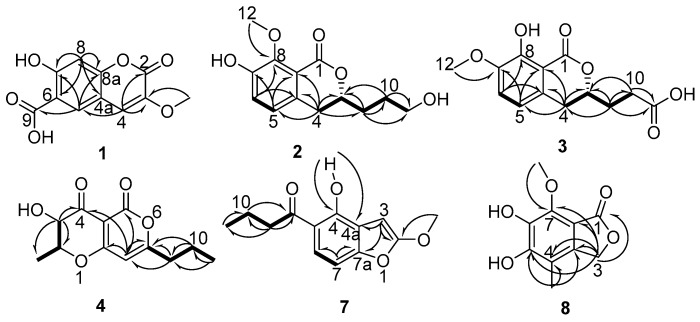
Key COSY (bold line) and HMBC (arrow) correlations of Compounds **1**–**4**, **7**, and **8**.

Compound **1** was isolated as a white solid with the molecular formula C_11_H_8_O_6_ established from HR-ESI-MS at *m/z* 235.0250 [M − H]^−^ (calcd for 235.0248, Figure S7). The ^1^H NMR (Table 1, Figure S1) showed two aromatic protons at δ_H_ 8.1 (H-5) and 6.9 (H-8), one olefin proton at δ_H_ 7.4 (H-4), and one methoxy group at δ_H_ 3.79 (H_3_-10). The ^13^C NMR (Table 1, Figure S2) and DEPT 135 spectra (Figure S3) of **1** displayed 11 carbon resonances assignable to one methoxy at δ_C_ 56.1 (C-10), three methines at δ_C_ 113.7 (C-4), 129.4 (C-5), and 103.3 (C-8), five quaternary carbons at δ_C_ 142.0 (C-3), 111.0 (C-6), 153.8 (C-7), 161.2 (C-8a), and 112.4 (C-4a), one *α*,*β*-unsaturated lactone carbonyl at δ_C_ 156.3 (C-2), and one carbonyl carbon at δ_C_ 171.1 (C-9). Detailed analysis of the 1D and 2D NMR spectra of **1** revealed that they were very similar to those of courmarin [13]. The HMBC (Figure 2, Figure S6) correlations H-4/C-2, H-4/C-3, H-4/C-5, H-8/C-6, H-8/C-7, H-8/C-8a, H-8/C-4a, H-5/C-7, and H-5/C-8a established the coumarin core. The HMBC correlations H-4/C-3 and Me-10/C-3 revealed that the methoxyl group was located at C-3. Furthermore, the HMBC correlations H-5/C-4 and H-5/C-9 showed that the carboxyl functional group attached at C-6. The HMBC correlations H-5/C-7, H-5/C-8a, H-8/C-7, and H-8/C-8a supported the presence of a coumarin derivative with a hydroxyl group at C-7. Combined with HSQC (Figure S5) and HMBC, Compound **1** was determined as shown (Figure 1).

**Figure 3:**
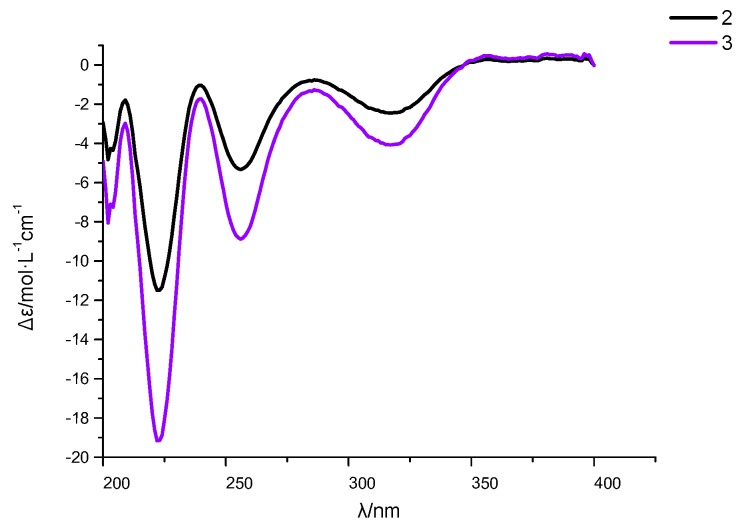
ECD spectra of Compounds **2** and **3**.

Compound **2** was assigned the molecular formula C_13_H_16_O_5_ by HR-ESI-MS at *m/z* 251.0930 [M − H]^−^ (calcd for 251.0925, Figure S13). The ^1^H NMR (Table 1, Figure S8) exhibited two aromatic proton resonances at δ_H_ 6.95 (H-5) and 7.10 (H-6), indicating the presence of a 1,2,3,4-tetrasubstituted phenyl. The ^1^H NMR data displayed signals for one oxygenated methine proton signal at δ_H_ 4.48 (H-3), three methylene proton signals at δ_H_ 2.88 (H-4), 1.83 (H-9), and 1.69 (H-10), one oxymethylene proton signal at δ_H_ 3.63 (H-11), and one methoxy group at δ_H_ 3.87 (H-12). The ^13^C NMR (Table 1) and DEPT (Figure S9) spectra displayed 13 signals for six aromatic carbons at δ_C_ 133.2 (C-4a), 124.3 (C-5), 123.0 (C-6), 151.5 (C-7), 150.5 (C-8), and 119.4 (C-8a), four methylenes at δ_C_ 34.3 (C-4), 32.2 (C-9), 29.1 (C-10), and 62.5 (C-11), one methine at δ_C_ 80.3 (C-3), a carbonyl carbon at δ_C_ 165.2 (C-1), and one methoxy group at δ_C_ 61.9 (C-12). The above spectroscopic features suggested that **2** belongs to the dihydroisocoumarins class. Further analysis of the HMBC spectrum (Figure 2, Figure S12), especially the presence of the correlations OMe-12/C-8, H-4/C-5, H-4/C-4a, H-4/C-8a, H-4/C-3, H-4/C-9, H-5/C-8a, H-5/C-7, H-6/C-4a, and H-6/C-8 suggested a dihydroisocoumarins derivative with a methoxy group at C-8 and a hydroxyl group at C-7. Obvious differences were inferred from the presence of a methoxy group at C-8 in Compound **2** and at C-7 in peniciisocoumarin C [14]. Furthermore, the COSY (Figure 2, Figure S10) correlations H-4/H-3/H-9/H-10/H-11 and the HMBC (Figure S12) correlations H-9/C-3, H-9/C-4, H-9/C-10, H-9/C-11, H-10/C-3, and H-10/C-11 indicated the presence of the fragment –CH_2_–CH–CH_2_–CH_2_–CH_2_–. Therefore, the structure of Compound **2** was elucidated as 7-hydroxy-3-(3-hydroxypropyl)-8-methoxyisochroman-1-one. The absolute configuration of C-3 was determined by CD. The negative circular dichroism at 258 nm (Figure 3), by comparison with data for dihydroisocoumarins described in the literature, suggested an *R* configuration at C-3 [15]. Thus, the absolute configuration of **2** was identified as being 3*R*.

Compound **3** was obtained as a white solid, having the molecular formula C_13_H_14_O_6_ based on the HR-ESI-MS at *m/z* 265.0716 [M − H]^−^ (calcd for 265.0712, Figure S19). The ^1^H NMR (Table 1, Figure S14) and HSQC (Figure S17) revealed two aromatic protons at δ_H_ 6.94 (H-5) and 7.10 (H-6), one oxymethine proton signal at δ_H_ 4.50 (H-3), three methylene proton signals at δ_H_ 2.88 (H-4), 2.05 (H-9), and 2.54 (H-10), and one methoxy group at δ_H_ 3.86 (H-12). The ^13^C NMR (Table 1, Figure S15) and DEPT data indicated that Compound **3** also shared the same dihydroisocoumarins skeleton. In addition, these spectroscopic features suggested that **3** was closely related to the known compound peniciisocoumarin C [14]. The obvious difference was that one oxymethylene group at C-10 in peniciisocoumarin C had been replaced by a carboxyl group at C-10 in Compound **3**. The COSY spectrum (Figure 2, Figure S16) revealed ^1^H–^1^H spin systems of H-4/H-3/H-9/H-10, allowing for the assignment of the fragments –CH_2_–CH–CH_2_–CH_2_–. The HMBC (Figure 2, Figure S18) correlations H-9/C-10, H-9/C-11, H-9/C-4, H-9/C-3, H-10/C-3, and H-10/C-11 supported the above deduction. The HMBC correlations H-12/C-7, H-5/C-4, H-5/C-7, H-5/C-8a, H-6/C-8, and H-6/C-4a indicated a methoxy group located at C-7 and a hydroxyl group at C-8. The absolute configuration of C-3 was determined to be *R* by CD spectroscopy (Figure 3) [14]. Thus, Compound **3** was named as (*R*)-3-(8-hydroxy-7-methoxy-1-oxoisochroman-3-yl)-propanoic acid. 

**Figure 4:**
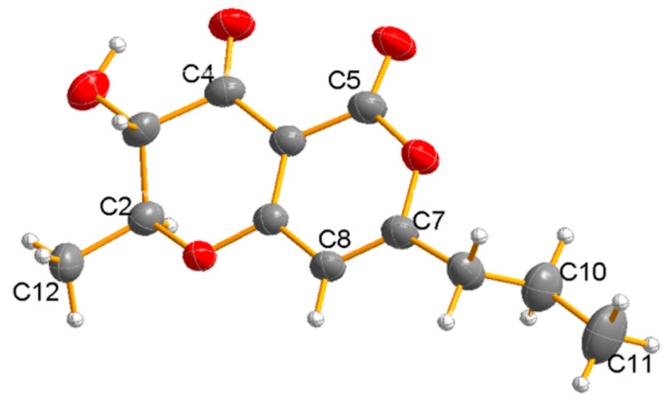
*X*-ray structure of **4**.

Compound **4** was obtained as a white solid. The molecular formula was determined as C_12_ H_14_ O_5_ by HR-ESI-MS ion at *m/z* 237.0771 [M − H]^−^ (calcd for 237.0769, Figure S25). The ^1^H NMR (Table 2, Figure S20) spectrum of **4** showed signals of one aromatic proton at δ_H_ 5.90 (H-8), two methyls at δ_H_ 0.98 (H_3_-11) and 1.64 (H_3_-12), two methylenes at δ_H_ 1.71 (H_2_-10) and 2.48 (H_2_-9), and two methines at δ_H_ 3.99 (H-3) and 4.36 (H-2). The ^13^C NMR data (Table 2, Figure S21) of **4** revealed 12 carbon resonances assignable to two methyls at δ_C_ 13.5 (C-11) and 18.2 (C-12), two methylenes at δ_C_ 36.5 (C-9) and 19.9 (C-10), three methines at δ_C_ 80.2 (C-2), 99.1 (C-8), and 72.1 (C-3), three quaternary carbons at δ_C_ 97.6 (C-4a), 176.5 (C-8a), and 173.5 (C-7), and two carbonyl carbons at δ_C_ 188.9 (C-4) and 157.8 (C-5). Detailed analysis of the 1D and 2D NMR spectra of **4** revealed that they were very similar to those of radicinin [12] with a difference at the single bond between C-9 and C-10 in **4** instead of a double bond in radicinin. The ^1^H–^1^H COSY spectrum (Figure 2, Figure S22) revealed ^1^H–^1^H spin systems of H-9/H-10/H-11 and H-2/H-3/H-12, allowing for an assignment of the fragments –CH_2_–CH_2_–CH_3_ and CH_3_–CH–CH–. The HMBC (Figure 2, Figure S24) correlations H-9/C-7, H-9/C-8, and H-10/C-7 indicated that the propyl unit was connected at C-7. Furthermore, the correlations from H-3/C-2, H-3/C-4, and H-3/C-12 showed the presence of a methyl at C-2 and a hydroxyl group at C-3. The correlation signal of the ^1^H–^1^H COSY spectrum also supported the above deduction. Therefore, the structure of Compound **4** was elucidated as 3-hydroxy-2-methyl-7-propyl-2, 3-dihydropyrano [4,3-b]pyran-4, and 5-dione. The absolute configuration of C-2 and C-3 was determined to be 2*S* and 3*S* by the X-ray diffraction analysis of a single crystal using Cu Kα (Figure 4). Compound **4** has been reported as a derivative of radicinin by semi-synthesis in [12] and here is reported as a natural fungal product for the first time, identified as being (2*S*, 3*S*)-3-hydroxy-2-methyl-7-propyl-2, 3-dihydropyrano [4,3-b]pyran-4,5-dione, named dihydroradicinin.

Compound **7** was isolated as a white powder. Its molecular formula was deduced as C_13_H_14_O_4_ based on the HR-ESI-MS (*m/z* 233.0821 [M − H]^−^ (calcd for 233.0819), Figure S31) and NMR data, implying seven degrees of unsaturation. The ^1^H NMR data (Table 2, Figure S26) of **7** displayed signals of one phenolic hydroxy proton at δ_H_ 15.11 (OH-4), two aromatic protons at δ_H_ 7.09 (H-6) and 7.15 (H-7), one olefin proton at δ_H_ 6.21 (H-3), one methyl at δ_H_ 0.96 (H-11), two methylenes at δ_H_ 1.67 (H-10) and 2.98 (H-9), and one methoxyl group at δ_H_ 3.91 (H_3_-12). The ^13^C NMR data (Table 2, Figure S27) of **7** exhibited 13 carbon resonances assignable to one methyl at δ_C_ 14.2 (C-11), one methoxy at δ_C_ 56.2 (OMe-12), two methylenes at δ_C_ 46.7 (C-9) and 18.8 (C-10), three sp^2^ methines at δ_C_ 91.5 (C-3), 119.8 (C-7), and 124.3 (C-6), five quaternary carbons at δ_C_ 162.9 (C-2), 105.5 (C-4a), 166.2 (C-4), 104.1 (C-5), and 163.5 (C-7a), and one carbonyl carbon at δ_C_ 206 (C-8). The COSY (Figure 2, Figure S28) correlation between H-9/H-10/H-11 and the HMBC correlations between H-9/C-8, H-9/C-10, H-9/C-11, H-10/C-8, and H-10/C-11 indicated the presence of the fragment CH_3_–CH_2_–CH_2_–. The structure of Compound **7** was further confirmed by the HMBC data. The observation of the HMBC (Figure 2, Figure S30) correlations H-3/C-4a, H-3/C-2, H-3/C-7a, H-7/C-6, and H-7/C-7a constructed a benzofuran skeleton. The HMBC correlations from OMe-12/C-2, OH-4/C-4a, and OH-4/C-4 indicated that a methoxy group was attached at C-2 and that a hydroxyl group was connected at C-4. On the basis of the above data, the structure of Compound **7** was determined as 1-(4-hydroxy-2-methoxybenzofuran-5-yl) butan-1-one.

Compound **8** was obtained as a colorless crystal, of which the molecular formula was established as C_10_H_10_O_5_ based on HR-ESI-MS at *m/z* 209.0456 [M − H]^−^ (calcd for 209.0455, Figure S37), which was in agreement with the ^1^H and ^13^C-NMR spectra. The ^1^H-NMR spectrum (Table 2, Figure S32) of **8** suggested signals attributable to a methoxyl group at δ_H_ 3.80 (H_3_-9), a methylene at δ_H_ 5.17 (H_2_-3), and a methyl group at δ_H_ 2.06 (H_3_-8). Analyses of the ^13^C NMR (Table 2, Figure S33) and DEPT 135 spectrum data revealed the presence of 10 carbons, including three sp^3^-carbon signals related to a methoxyl group at δ_C_ 61.2 (C-9), an oxymethylene moiety at δ_C_ 70.1 (C-3), a methyl group at δ_C_ 10.7 (C-8), and seven quaternary sp^2^-carbon atoms, including a carboxy carbon signal at δ_C_ 173.4 (C-1) and six aromatic quaternary carbon signals at δ_C_ 103.9 (C-7a), 111.4 (C-4), 136.6 (C-7), 143.8(C-4a), 149.2 (C-6), and 156.7 (C-5). The protonated carbon atoms and their corresponding protons and the full connection of Compound **8** were established by using HSQC (Figure S35) and HMBC experiments, respectively. The HMBC (Figure 2, Figure S36) correlations from H_3_-8/C-4, H_3_-8/C-4a, H_3_-8/C-5, and H_2_-3/C-4 showed that the methyl was connected at C-4 and a hydroxyl group was located at C-5. Moreover, the weak correlations from H_2_-3/C-5, H_2_-3/C-7, and H-9/C-7 indicated a methoxyl group located at C-7. The ^1^H and ^13^C NMR spectra of **8** were similar to those of epicoccone B [16], except for the presence of the methoxy group at C-7. Thus, the structure of **8** was elucidated as being 5,6-dihydroxy-7-methoxy-4-methylisobenzofuran-1(3H)-one.

In addition, the structures of 3-epideoxyradicinol (**5**) [17], the radicinol derivative (**6**) [18], 4,6-dihydroxy-5-methoxy-7-methylphthalide (**9**) [19], 4,5,6-trihydroxy-7-methyl phthalide (**10**) [19], epicoccone B (**11**) [16], 4,6-dihydroxy-5-methoxy-7-methyl-1,3-dihydroisobenzofuran (**12**) [20], and 4,5,6-trihydroxy-7-methyl-1,3-dihydroisobenzofuran (**13**) [20] were determined by comparing the NMR data with those reported in the literature. 

Accordingly, Compounds **1**–**13** were assayed for their antimicrobial activity against five bacteria (*S. aureus* (ATCC 6538), *B. subtilis* (ATCC 6633), *E. coli* (ATCC 8739), *P. aeruginosa* (ATCC 9027), and *S. enteritidis* (ATCC 14028)) along with three phytopathogenic fungi (*P. italicum* (BNCC 118157)*, C. musae* (BNCC 226680), and *G. zeae (*BNCC 116158)) for the first time. The results disclosed that Compounds **6** and **7** showed antibacterial activities with the MIC values between 25 and 50 μg/mL against *B. subtilis* (ATCC 6538), *E. coli* (ATCC 8739), and *S. aureus* (ATCC 6538) (Table 3). All compounds showed no significant activity against phytopathogenic fungi at 100 μg/mL. Meanwhile, the antioxidant activity test using DPPH free radicals indicated that Compounds **10**–**13** showed potent antioxidant activity (Table 4) with IC_50_ values of 13.6, 12.1, 18.1, and 11.7 μg/mL, respectively. It is noteworthy that the antioxidant activity was evaluated for the first time for Compound **11**. Isocoumarins and benzofuranones from natural sources are excellent antioxidants, antitumor, and antimicrobe agents [21,22,23,24]. However, no fungal species from genus *Epicoccum* have been reported to produce antioxidant metabolites. Antioxidants may be a promising prevention or therapeutic intervention to help alleviate oxidative stress and to reduce the risk of many diseases [25,26,27,28,29]. For all compounds, the cytotoxic activity evaluation against MDA-MB-435, HepG2, A549, HCT116, and BT549 human cell lines exhibited no significant activity at 50 μM.

## 3. Experimental Section

### 3.1. General Experimental Procedures

HR-ESI-MS data were measured on a Thermo Fisher Scientific Q-TOF mass spectrometer (Thermo Fisher Scientific, Waltham, MA, USA). NMR spectra were performed on a Bruker AVANCE NEO 600 MHz spectrometer (Bruker BioSpin, Switzerland), using TMS as an internal standard. IR spectra were carried out on a Nicolet Nexus 670 spectrophotometer in KBr discs. CD spectra were measured on a ChirascanTM CD spectrometer (Applied Photophysics, London, UK). UV spectra were measured on a PERSEE TU-1990 spectrophotometer. Single-crystal data were recorded on an Oxford Gemini S Ultra diffractometer (Oxford Instrument, Oxfordshire, UK). Sephadex LH-20 (GE Healthcare Bio-Sciences AB, Stockholm, Sweden) column chromatography (CC) was carried out on silica gel (200–300 mesh, Qingdao Marine Chemical Factory, Qingdao, China). Thin layer chromatography (TLC) was detected on a silica gel GF254 plate (Qingdao Marine Chemical Ltd, Qingdao, China). A Phenomenex Luna (Phenomenex, Torrance, CA, USA) C18 column (250 × 10 mm, 5 μm, 5 mL/min) was used for semipreparative HPLC. All solvents were of analytical grade, except for those used for HPLC, which were of chromatographic grade. DPPH was purchased from Sigma (Steinheim, Germany).

### 3.2. Fungal Materials

The fungus SCNU-F0002 used in the study was isolated from fresh fruit of the mangrove plant *Acanthus ilicifolius* L., which was collected in January 2018 from the Qi’ao island Mangrove Nature Reserve in Guangdong province, China. It was obtained using the standard protocol for isolation [30]. Initially, the plant fruit was washed with sterile water and surface-sterilized in a 100 mL beaker with 75% ethanol for 1 min. This was followed by dipping the sample into a 5% sodium hypochlorite for 1 min, and the plant parts were then rinsed with sterile water, cut into 3 mm sections, and plated on potato dextrose agar (PDA) (potatoes: 300 mg/mL; dextrose: 20 mg/mL; agar: 15 mg/mL; chloramphenicol: 1 mg/mL) with penicillin (100 units/mL) and streptomycin (0.8 mg/mL). The plates were incubated at 25 ± 1 °C. The endophytic fungal strains were isolated by routine microbiology. The fungal isolates were numbered and stored at 4 °C in triplicate on PDA slants. Fungal identification was carried out using a molecular biological protocol by DNA amplification and sequencing of the ITS region [31]. The sequence data of the fungal strain have been deposited at Gen Bank with accession no. MN096740. A BLAST search result showed that the sequence was most similar (100%) to the sequence of *Epicoccum nigrum*. A voucher strain was deposited at the School of Chemistry and Environment, South China Normal University, Guangzhou, China, with the access code SCNU-F0002.

### 3.3. Extraction and Isolation 

The fungus *Epicoccum nigrum* SCNU-F0002 was grown under static conditions at 25 °C for 28 days in a solid autoclaved rice substrate medium containing 50 g of rice and 50 mL of 3‰ saline water. After incubation, the mycelia and solid rice were extracted with EtOAc, and the extracts were concentrated to yield 22.5 g of residue under reduced pressure. The residue was then subjected to a silica gel column (80 × 10 cm) and eluted with a gradient of petroleum ether/EtOAc from 1:0 to 0:1 and divided into 36 fractions. Fraction 10 (115 mg) was chromatographed on Sephadex LH-20 CC (CH_2_Cl_2_/MeOH *v*/*v*, 1:1) and silica gel CC (petroleum ether/EtOAc, *v*/*v*, 1:5) gave Compounds 4 (7 mg), 5 (5 mg), and 7 (10 mg). Fraction 18 (600 mg) was further eluted on silica gel CC (CH_2_Cl_2_/MeOH *v*/*v*, 100:2) to yield Compounds **1** (4 mg), **6** (5.1 mg), and **8** (6.2 mg). Fraction 28 (240 mg) was purified via the Sephadex LH-20 CC (CH_2_Cl_2_/MeOH *v*/*v*, 3:1) to yield Subfraction 28.5 (30 mg), which was purified by silica gel CC (CH_2_Cl_2_/MeOH *v*/*v*, 96:4) to afford Compounds **2** (3.8 mg), **9** (6 mg), and **10** (5.3 mg). Fraction 30 (12 mg) was purified by semipreparative RP-HPLC (70% acetonitrile/H_2_O) to yield **3** (5 mg, *t*_R_ = 18.3 min). Fraction 33 (80 mg) was eluted on silica gel CC (petroleum ether/EtOAc *v*/*v*, 3:1) to yield six fractions (subfractions 33.1–33.6). Subfraction 33.3 (43 mg) was applied to Sephadex LH-20 CC (CH_2_Cl_2_/MeOH *v*/*v*, 1:1) to furnish Compounds **11** (9.6 mg), **12** (10 mg), and **13** (3.8 mg).

Compound **1**: white solid; UV (MeOH) λmax (logε): 368 (7.23) nm; IR (KBr) νmax: 3400, 1630, 1140, 820, 710 cm^−1^; ^1^H and ^13^C NMR data, see Table 1; HR-ESI-MS *m/z* 235.0250 [M − H]^−^ (calcd for 235.0248).

Compound **2**: white solid; ${\left[\alpha \right]}_{D}^{25}$ −12.3 (*c* 0.12, MeOH); UV (MeOH) λmax (logε): 219 (6.52), 336 (4.56) nm; IR (KBr) νmax: 3430, 2960, 2920; 2870, 1750, 1650, 1520, 1459, 1370, 1260, 1050, 972, 876, 804, 702, 638 cm^−1^; ^1^H and ^13^C NMR data, see Table 1; HR-ESI-MS *m/z* 251.0930 [M − H]^−^ (calcd for 251.0925).

Compound **3**: white solid; ${\left[\alpha \right]}_{D}^{25}$ −15.3 (*c* 0.15, MeOH); UV (MeOH) λmax (logε): 336 (3.56) nm; IR (KBr) νmax: 3471, 3220, 2926, 2872, 1648, 1596, 1486, 1284, 1223, 1135, 1060, 945, 809, 702, 654 cm^−1^; ^1^H and ^13^C NMR data, see Table 1; HR-ESI-MS *m/z* 265.0716 [M − H]^−^ (calcd for 265.0712).

Compound **4**: white solid; ${\left[\alpha \right]}_{D}^{25}$ −8.2 (*c* 0.15, MeOH); UV (MeOH) λmax (logε): 315 (10) nm; IR (KBr) νmax: 3075, 3000, 2935, 1742, 1650, 1550, 1115 cm^−1^; ^1^H and ^13^C NMR data, see Table 2; HR-ESI-MS *m/z* 237.0771 [M − H]^−^ (calcd for 237.0769).

Compound **7**: white powder; UV (MeOH) λmax (logε): 296 (1.20) nm; IR (KBr) νmax: 3276, 2934, 1578, 1446, 1215,1095, 820 cm^−1^; ^1^H and ^13^C NMR data, see Table 2; HR-ESI-MS *m/z* 233.0821 [M − H]^−^ (calcd for 233.0819).

Compound **8**: colorless crystal; UV (MeOH) λmax (logε): 299 (1.734) nm; IR (KBr) νmax: 3388, 1620, 1163, 1006, 785, 675 cm^−1^; ^1^H and ^13^C NMR data, see Table 2; HR-ESI-MS *m/z* 209.0456 [M − H]^−^ (calcd for 209.0455).

X-ray crystal data for **4**. Colorless crystals of **4** were obtained in methanol. Crystal data (CCDC 19011202) were collected with Cu Kα radiation. Monoclinic, space group P21 (no. 4), a = 4.61510(10) Å, b = 5.40930(10) Å, c = 23.3354(6) Å, *α* = 90, *β* = 93.637(2), *γ* = 90, V = 581.38(2) Å^3^, Z = 2, T = 199.99(10) K, *μ* (Cu Kα) = 0.897 mm^−1^, Dcalc = 1.361 g/cm3, F(000) = 252, R1 = 0.0413, wR2 = 0.1165. Crystal dimensions 0.4 × 0.3 × 0.02 mm^3^. Flack parameter = −0.06(13). The total number of independent reflections measured was 3935, of which 2145 were observed and collected in the range of 7.592° ≤ 2θ ≤ 146.412°. The structure was determined and refined using full-matrix least-squares on F2 values for 1.036 I > = 2*σ* (I).

To maximize the likelihood of success, a full sphere of data was collected using Cu Kα radiation. A total of 3935 reflections were collected, yielding a Flack parameter x and standard uncertainty *u* for this structure of −0.06 (13) based on 860 Friedel pairs. The value of *u* is slightly beyond the limit of enantiopure-sufficient distinguishing power. However, further confirmation of the absolute configuration was obtained from the examination of Bayesian statistics on Bijvoet pairs [32] implemented using the program *PLATON* [33]. The calculated Hooft y parameter was −0.06 (10) with *G* = 1.1 (1). The calculated probability values *P*3 (true), *P*3 (twin), and *P*3 (wrong) were 1.000, 0.000, and 0.000, respectively. This confirmed the absolute configuration of the two stereocenters as 2*S* and 3*S.* Moreover, these results are consistent with the relative configuration of radicinin that was proposed in [12,34] on the basis of NMR data and X-ray crystal data.

### 3.4. DPPH Radical Scavenging Activity Assay 

The DPPH free radical scavenging assay is based on previously reported methods [35,36], but with minor modifications. The assay was performed on a 96-well microplate. A total of 200 μL of the reaction mixture consists of a series of 100 μL of different concentrations (2, 25, 50, 100, 200 μg/mL) of the tested compound (in ethanol) and 100 μL of 0.16 mM DPPH (in ethanol). The reaction mixture was incubated for 30 min at room temperature in the dark. The absorbance at 517 nm was recorded on a microplate reader, and the inhibition rate was calculated. Vitamin C was used as a positive control.

### 3.5. Antimicrobial Activity Assay

The antimicrobial activities against five bacteria (*S. aureus* (ATCC 6538), *B. subtilis* (ATCC 6633), *E. coli* (ATCC 8739), *P. aeruginosa* (ATCC 9027), and *S. enteritidis* (ATCC 14028)) along with three phytopathogenic fungi (*P. italicum* (BNCC 118157), *C. musae* (BNCC 226680), and *G. zeae* (BNCC 116158)) were evaluated on 96-well microtiter plates using a modification of the broth microdilution method [37,38]. The microbial test strain was incubated, and the microbial strain suspension was diluted to a seeding density of 5 × 10^5^ cfu compared to the MacFarland standard. The fungi was cultured in PDB (potato dextrose broth) medium (6.75 g of potatoes, 0.45 g of dextrose, and 300 mL of distilled H_2_O) at 28 °C (160 rpm) for 48 h, and the bacteria was cultured in LB medium (3 g of peptone, 1.5 g of sodium chloride, 0.3 g of dextrose, 1.5 g of yeast extract, and 300 mL of distilled H_2_O) at 37 °C (160 rpm) for 24 h. Under the sterile environment, microorganism suspensions (100 μL) of each strain were poured into the wells containing 100 μL of 2-fold serially diluted single compounds in the corresponding culture medium for a final volume of 200 μL. The fungi and bacteria were then incubated at 28 °C for 48 h and at 37 °C for 24 h, respectively. The antimicrobial effect was evaluated by optical density measurement at 595 nm. The amount of growth in each well was compared with a blank control, which consisted only of the medium (an agent in DMSO and PDB), and the MIC was recorded as the lowest concentration of the agent that completely inhibits growth. All experiments were performed at least three times. Triadimefon and ciprofloxacin were used as positive controls for fungi and bacteria, respectively.

### 3.6. Cytotoxicity Assay

The cytotoxicities of Compounds **1**–**13** at a serial final concentration of 50, 25, 12.5, 6.25, and 3.125 μM were evaluated against A549 (human lung cancer), MDA-MB-435 (breast cancer cells), HepG2 (liver cancer cells), HCT116 (colon cancer cells), and BT549 (breast cancer cells) using the MTT method as described previously [39]. Human breast cancer cell lines MDA-MB-435 and BT549, human liver cancer cell line HepG2, human lung cancer cell line A549, and human colon cancer cell line HCT116 were obtained from Keygen Biotech (Nanjing, China) and cultured in Dulbecco’s modified Eagle’s medium (DMEM) (Invitrogen, Carlsbad, CA, USA) supplemented with 5% fetal bovine serum (Hyclone, Logan, UT, USA), 2 mM l-glutamine, 100 mg/mL streptomycin, and 100 units/mL penicillin (Invitrogen). The cultures were maintained at 37 °C in a humidified atmosphere of 5% CO_2_.

## 4. Conclusions

In summary, one new coumarin (**1**), two new isocoumarins (**2** and **3**), the naturally derived dihydroradicinin (**4**), and two new benzofuranone derivatives (**7** and **8**), together with seven known analogues (**5**–**6** and **9**–**13**) were isolated from the culture of the mangrove endophytic fungus *Epicoccum nigrum* SCNU-F0002. Structures of the new compounds were obtained by a detailed examination of their spectroscopic data, and their absolute configurations were obtained either by ECD spectra or by X-ray analysis. Compounds **10**–**13** showed potent anti-oxidative activity by DPPH radical-scavenging assay. Compounds **6** and **7** showed antibacterial effects against *Bacillus subtilis* (ATCC 6633)*, Escherichia coli* (ATCC 8739)*,* and *Staphylococcus aureus* (ATCC 6538) with the MIC values in the range of 25–50 μg/mL.

## Figures and Tables

**Table 1:** ^1^H (600 MHz) and ^13^C (150 MHz) NMR Data for **1**–**3**.

Position	1 ^a^		2 ^b^		3 ^b^	
	δ_C_	δ_H_(*J*/Hz)	δ_C_	δ_H_(*J*/Hz)	δ_C_	δ_H_(*J*/Hz)
1			165.2, C		164.9, C	
2	156.3, C					
3	142.0, C		80.3, CH	4.48, m	79.5, CH	4.50, m
4	113.7, CH	7.40, s	34.3, CH_2_	2.88, m	34.4, CH_2_	2.88, m
4a	112.4, C		133.2, C		133.0, C	
5	129.4, CH	8.10, s	124.3, CH	6.95, d (8.2)	124.3, CH	6.94, d (8.2)
6	111.0, C		123.0, CH	7.10, d (8.2)	123.0, CH	7.10, d (8.2)
7	153.8, C		151.5, C		150.5, C	
8	103.3, CH	6.90, s	150.5, C		151.5, C	
8a	161.2, C		119.4, C		119.3, C	
9	171.1, C		32.2, CH_2_	1.83, m	31.0, CH_2_	2.05, m
10	56.1, OCH_3_	3.79, s	29.1, CH_2_	1.69, m	30.6, CH_2_	2.54, m
11			62.5, CH_2_	3.63, t (6.0)	177.1, C	
12			61.9, OCH_3_	3.87, s	61.9, OCH_3_	3.86, s

^a^ Measured in DMSO-*d*_6_; ^b^ Measured in Methanol-*d*_4_.

**Table 2:** ^1^H (600 MHz) and ^13^C (150 MHz) NMR Data for **4** and **7**–**8**.

Position	4 ^a^		7 ^b^		8 ^c^	
	δ_C_	δ_H_(*J*/Hz)	δ_C_	δ_H_(*J*/Hz)	δ_C_	δ_H_(*J*/Hz)
1					173.4, C	
2	80.2, CH	4.36, dq (6, 12.6)	162.9, C			
3	72.1, CH	3.99, d (12.6)	91.5, CH	6.21, s	70.1, CH_2_	5.17, s
4	188.9, C		166.2, C		111.4, C	
4a	97.6, C		105.5, C		143.8, C	
5	157.8, C		104.1, C		156.7, C	
6			124.3, CH	7.09, d (13.2)	149.2, C	
7	173.5, C		119.8, CH	7.15, d (13.2)	136.6, C	
7a			163.5, C		103.9, C	
8	99.1, CH	5.90, s	206, C		10.7, CH_3_	2.06, s
8a	176.5, C					
9	36.5, CH_2_	2.48, t (7.2)	46.7, CH_2_	2.98, t (7.2)	61.2, OCH_3_	3.80, s
10	19.9, CH_2_	1.71, qt (7.2, 7.8)	18.8, CH_2_	1.67, qt (7.2, 7.8)		
11	13.5, CH_3_	0.98, t (7.8)	14.2, CH_3_	0.96, t (7.8)		
12	18.2, CH_3_	1.64, d (6)	56.2, OCH_3_	3.91, s		
4-OH				15.11, s		

^a^ Measured in CDCl_3_. ^b^ Measured in acetone-*d*_6_. ^c^ Measured in Methanol-*d*_4_.

**Table 3:** Antibacterial activities of Compounds **1**–**13**.

	Strains	MIC (μg/mL)				
Compounds ^a^		*S. aureus* (G+)	*B. subtilis* (G+)	*E. coli* (G−)	*P. aeruginosa* (G−)	*S. enteritidis* (G−)
6		>100	50	>100	>100	>100
7		>100	25	50	>100	>100
Ciprofloxacin ^b^		0.25	0.25	0.5	0.5	0.25

^a^ Compounds **1**–**5** and **8**–**13** showed no activity (MIC > 1 mg/mL). ^b^ Positive control. G+: Gram-positive bacteria. G−: Gram-negative bacteria.

**Table 4:** DPPH free radical scavenging activities of Compounds **1**–**13**.

Compound	1	2	3	4	5	6	7	8	9	10	11	12	13	Vitamin C ^a^
IC_50_(μg/mL)	-	-	-	-	-	-	-	-	62.9	13.6	12.1	18.1	11.7	18.2

-: no activity (IC_50_ > 100 μg/mL); ^a^ positive control.

## Supplementary Materials

The following are available online at https://www.mdpi.com/1660-3397/17/7/414/s1, the Figure S1: ^1^H-NMR spectrum of compound (**1**), Figure S2: ^13^C-NMR spectrum of compound (1), Figure S3: DEPT 135 spectrum of compound (**1**), Figure S4: ^1^H-^1^H COSY spectrum of compound (**1**), Figure S5: HSQC spectrum of compound (**1**), Figure S6: HMBC spectrum of compound (**1**), Figure S7: HR-ESI-MS spectrum of compound (**1**), Figure S8: ^1^H-NMR spectrum of compound (**2**), Figure S9: DEPT 135, DEPT 90 and 13C-NMR spectrum of compound (**2**), Figure S10: ^1^H-^1^H COSY spectrum of compound (**2**), Figure S11: HSQC spectrum of compound (**2**), Figure S12: HMBC spectrum of compound (**2**), Figure S13: HR-ESI-MS spectrum of compound (**2**), Figure S14: ^1^H-NMR spectrum of compound (**3**), Figure S15. DEPT 135, DEPT 90 and ^13^C-NMR spectrum of compound (3), Figure S16: ^1^H-^1^H COSY spectrum of compound (**3**), Figure S17: HSQC spectrum of compound (**3**), Figure S18: HMBC spectrum of compound (**3**), Figure S19: HR-ESI-MS spectrum of compound (**3**), Figure S20: ^1^H-NMR spectrum of compound (**4**), Figure S21: ^13^C-NMR spectrum of compound (**4**), Figure S22: ^1^H-^1^H COSY spectrum of compound (**4**), Figure S23: HSQC spectrum of compound (**4**), Figure S24: HMBC spectrum of compound (**4**), Figure S25: HR-ESI-MS spectrum of compound (**4**), Figure S26: ^1^H-NMR spectrum of compound (**7**), Figure S27: ^13^C-NMR spectrum of compound (**7**), Figure S28: ^1^H-^1^H COSY spectrum of compound (**7**), Figure S29: HSQC spectrum of compound (**7**), Figure S30: HMBC spectrum of compound (**7**), Figure S31: HR-ESI-MS spectrum of compound (**7**), Figure S32: ^1^H-NMR spectrum of compound (**8**), Figure S33: ^13^C-NMR spectrum of compound (**8**), Figure S34: DEPT 135 spectrum of compound (**8**), Figure S35: HSQC spectrum of compound (**8**), Figure S36: HMBC spectrum of compound (**8**), Figure S37: HR-ESI-MS spectrum of compound (**8**).
