# Supplementary material for: The Purification, Characterization, and Biological Activity of New Polyketides from Mangrove-Derived Endophytic Fungus Epicoccum nigrum SCNU-F0002
Source: Mar Drugs. 2019 Jul 12;17(7):414. doi: 10.3390/md17070414 (PMC6669579; doi:10.3390/md17070414)
Supplement: Supplementary file 1 [file marinedrugs-17-00414-s001.pdf]

Supporting Information

Purification、Characterization and Biological Activity of New Polyketides from Mangrove Endophytic Fungus Epicoccum nigrum SCNU-F0002

Zhangyuan Yan[1], Shitong Wen[1], Meng Ding[2], Huixian Guo[1], Cuiying Huang[1], Xintong Zhu[1], Junyi Huang[1], Zhigang She[2], Yuhua Long [1*]

[1] School of Chemistry and Environment, South China Normal University, Guangzhou 510006, PR China; yzy2016edu@163.com (Z.Y.); 179694543@qq.com (S.W.); 1071238034@qq.com (H.G.); 1306872452@qq.com (C.H.); 390779509@qq.com (X.Z); 1097527770@qq.com (J.H.)

[2] School of Chemistry, Sun Yat-Sen University, Guangzhou 510275, PR China; cesshzhg@mail.sysu.edu.cn (Z.S.); 302556596@qq.com (M.D.)

* Correspondence: yuhualong68@hotmail.com (Y.L.)

CONCENT

Fig.S1. ^{1}H- NMR spectrum (DMSO-d_6, 600MHz) of compound (1) 3
Fig.S2. ^{13}C- NMR spectrum (DMSO-d_6, 150MHz) of compound (1) 3
Fig.S3. DEPT 135 spectrum (DMSO-d_6, 150MHz) of compound (1) 4
Fig.S4.^{1}H-^{1}H COSY spectrum (DMSO-d_6, 600MHz) of compound (1) 4
Fig.S5. HSQC spectrum (DMSO-d_6, 600MHz) of compound (1) 5
Fig.S6. HMBC spectrum (DMSO-d_6, 600MHz) of compound (1) 5
Fig.S7. HR-ESI-MS spectrum of compound (1) 6
Fig.S8. ^{1}H- NMR spectrum (CD_3OD, 600MHz) of compound (2) 6
Fig.S9. DEPT 135, DEPT 90 and ^{13}C-NMR spectrum (CD_3OD, 150MHz) of compound (2) 7
Fig.S10. ^{1}H-^{1}H COSY spectrum (CD_3OD, 600MHz) of compound (2) 7
Fig.S11. HSQC spectrum (CD_3OD, 600MHz) of compound (2) 8
Fig.S12. HMBC spectrum (CD_3OD, 600MHz) of compound (2) 8
Fig.S13. HR-ESI-MS spectrum of compound (2) 9
Fig.S14. ^{1}H- NMR spectrum (CD_3OD, 600MHz) of compound (3) 9
Fig.S15. DEPT 135, DEPT 90 and ^{13}C-NMR spectrum (CD_3OD, 150MHz) of compound (3) 10
Fig.S16. ^{1}H-^{1}H COSY spectrum (CD_3OD, 600MHz) of compound (3) 10
Fig.S17. HSQC spectrum (CD_3OD, 600MHz) of compound (3) 11
Fig.S18. HMBC spectrum (CD_3OD, 600MHz) of compound (3) 11
Fig.S19. HR-ESI-MS spectrum of compound (3) 12
Fig.S20. ^{1}H- NMR spectrum ($CDCl_3$, 600MHz) of compound (4) 12
Fig.S21. ^{13}C- NMR spectrum ($CDCl_3$, 150MHz) of compound (4) 13
Fig.S22. ^{1}H-^{1}H COSY spectrum ($CDCl_3$, 600MHz) of compound (4) 13
Fig.S23. HSQC spectrum ($CDCl_3$, 600MHz) of compound (4) 14
Fig.S24. HMBC spectrum ($CDCl_3$, 600MHz) of compound (4) 14
Fig.S25. HR-ESI-MS spectrum of compound (4) 15
Fig.S26. ^{1}H- NMR spectrum (acetone-d_6, 600MHz) of compound (7) 15
Fig.S27. ^{13}C- NMR spectrum (acetone-d_6, 150MHz) of compound (7) 16
Fig.S28. ^{1}H-^{1}H COSY spectrum (acetone-d_6, 600MHz) of compound (7) 16
Fig.S29. HSQC spectrum (acetone-d_6, 600MHz) of compound (7) 17
Fig.S30. HMBC spectrum (acetone-d_6, 600MHz) of compound (7) 17
Fig.S31. HR-ESI-MS spectrum of compound (7) 18
Fig.S32. ^{1}H- NMR spectrum (CD_3OD, 600MHz) of compound (8) 18
Fig.S33. ^{13}C- NMR spectrum (CD_3OD, 150MHz) of compound (8) 19
Fig.S34. DEPT 135 spectrum (CD_3OD, 150MHz) of compound (8) 19
Fig.S35. HSQC spectrum (600MHz, CD_3OD) of compound (8) 20
Fig.S36. HMBC spectrum (600MHz, CD_3OD) of compound (8) 20
Fig.S37. HR-ESI-MS spectrum of compound (8) 21

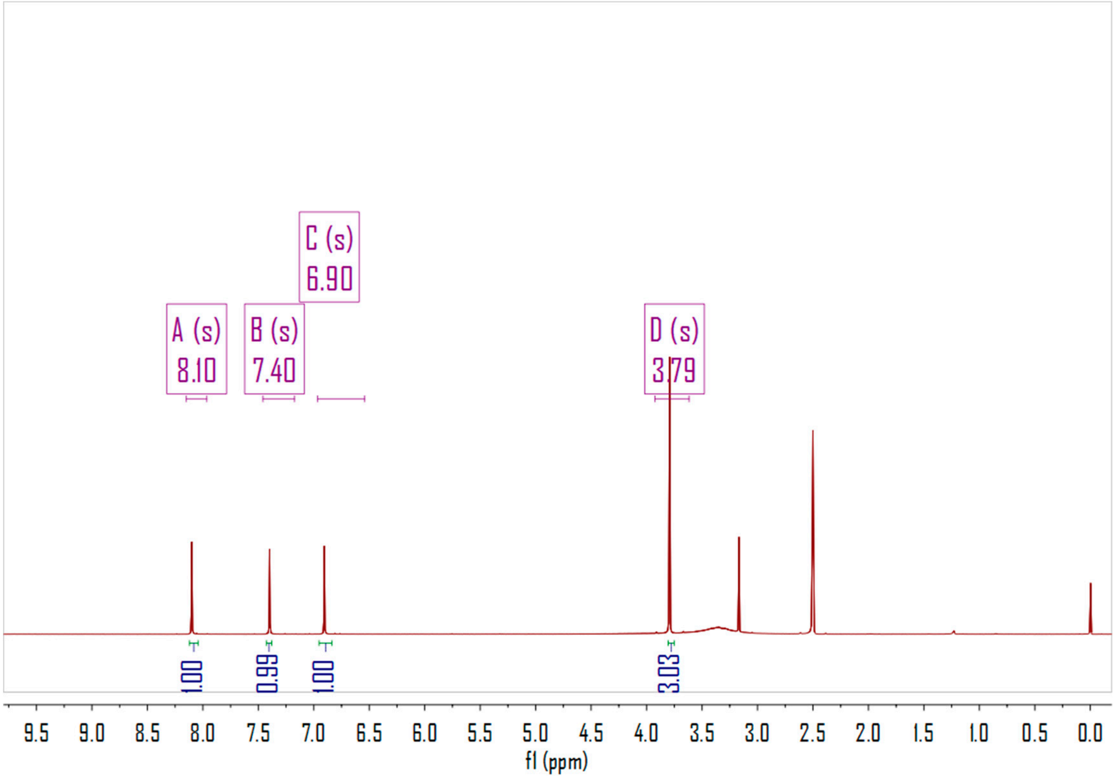

Fig.S1. ^{1}H- NMR spectrum (DMSO-d_6, 600MHz) of compound (**1**)

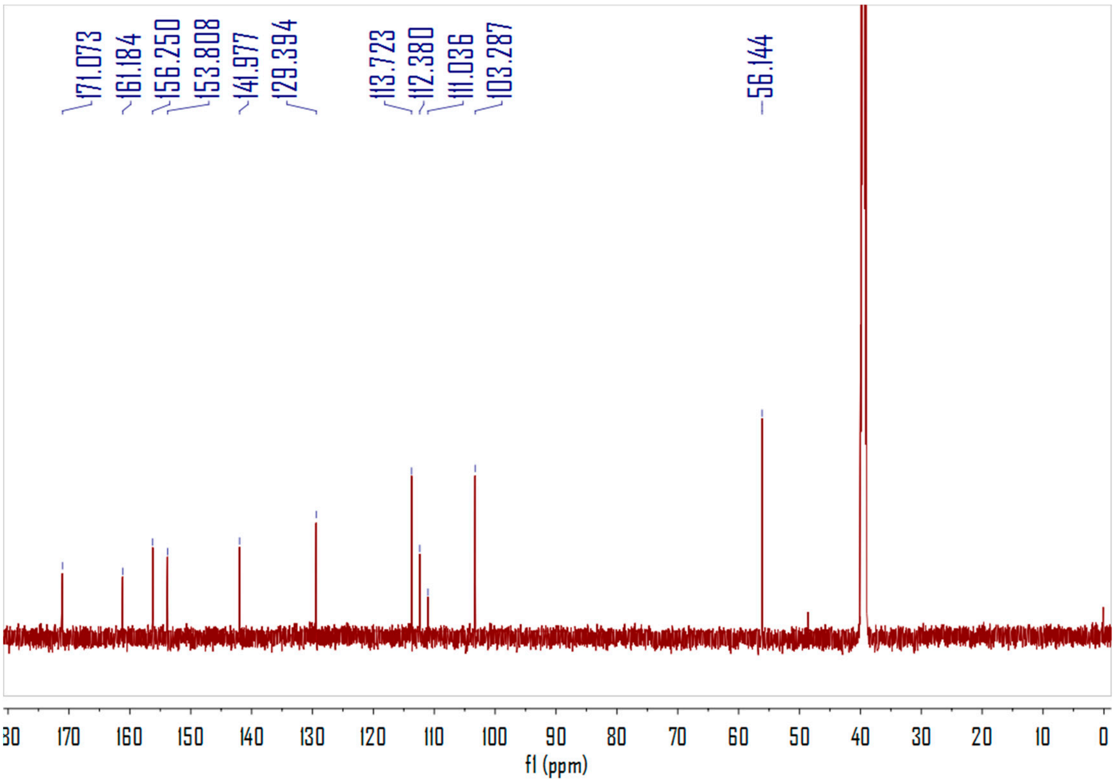

Fig.S2. ^{13}C- NMR spectrum (DMSO-d_6, 150MHz) of compound (**1**)

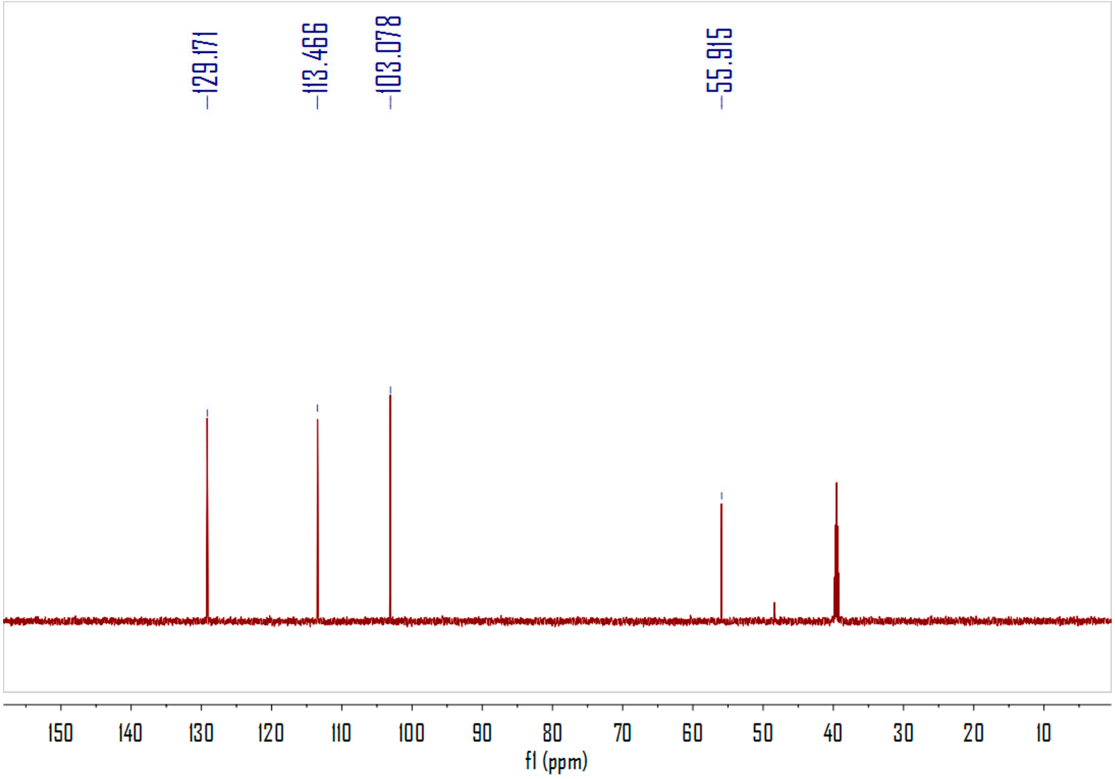

Fig.S3. DEPT 135 spectrum (DMSO-d_6, 150MHz) of compound (**1**)

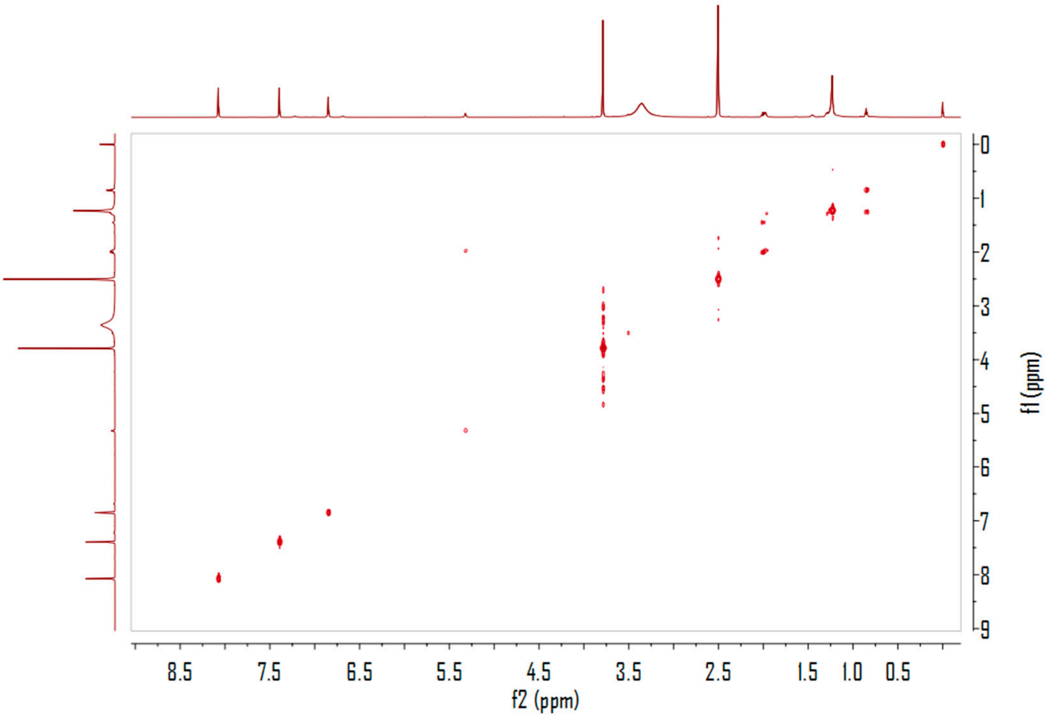

Fig.S4.^{1}H-^{1}H COSY spectrum (DMSO-d_6, 600MHz) of compound (**1**)

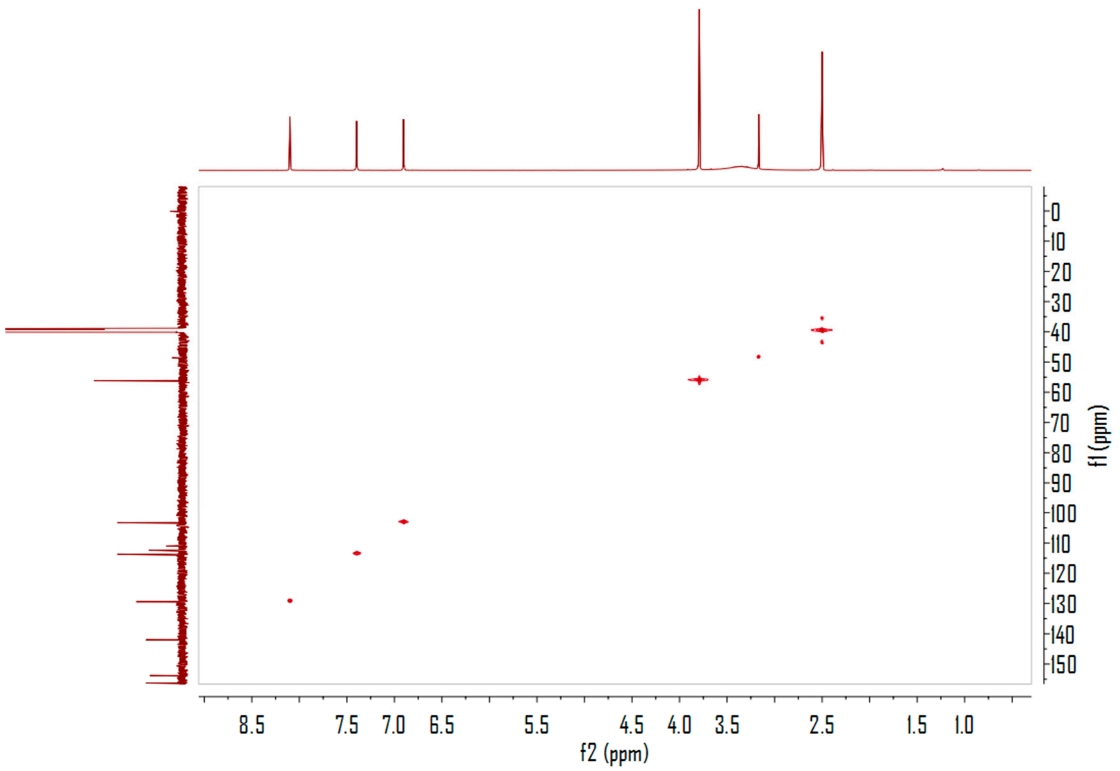

Fig.S5. HSQC spectrum (DMSO-d_6, 600MHz) of compound (**1**)

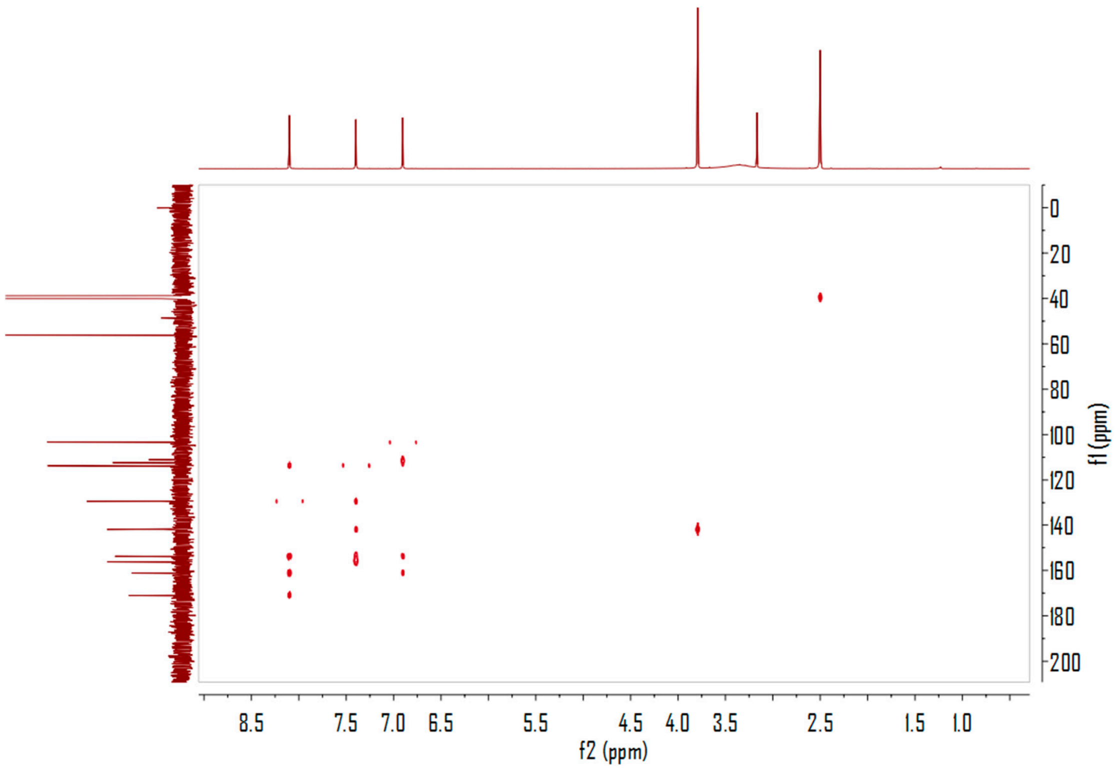

Fig.S6. HMBC spectrum (DMSO-d_6, 600MHz) of compound (**1**)

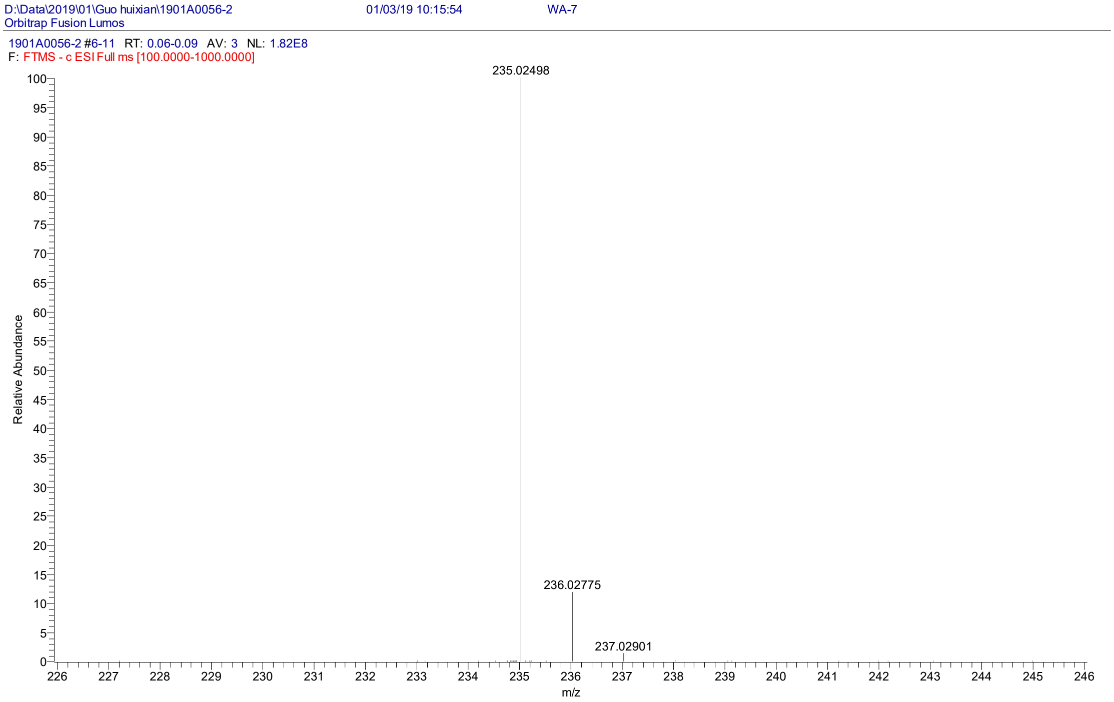

Fig.S7. HR-ESI-MS spectrum of compound (**1**)

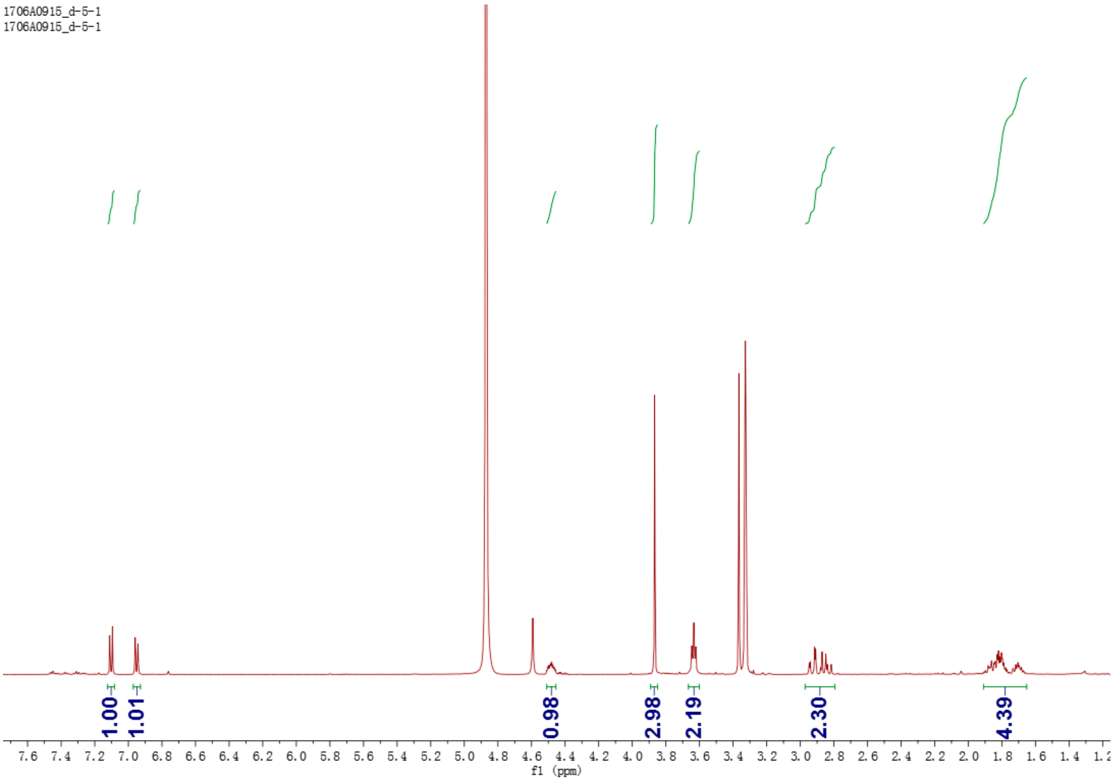

Fig.S8. ^{1}H- NMR spectrum (CD_3OD, 600MHz) of compound (**2**)

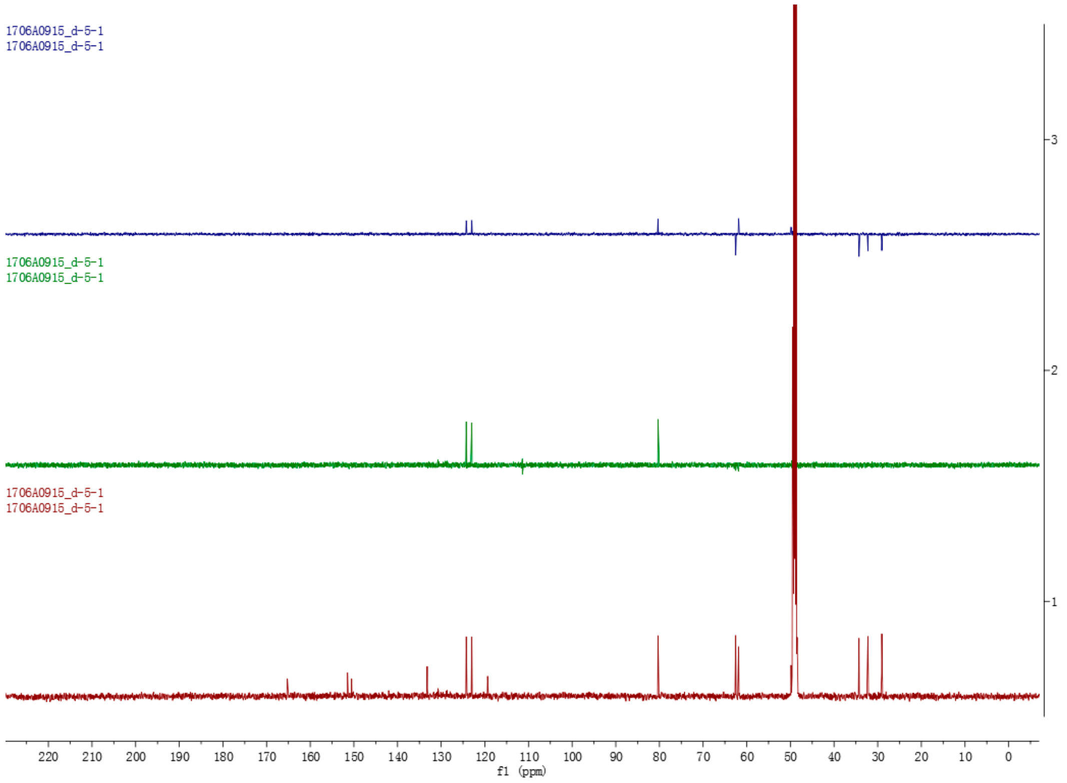

Fig.S9. DEPT 135, DEPT 90 and ^{13}C-NMR spectrum (CD_3OD, 150MHz) of compound (**2**)

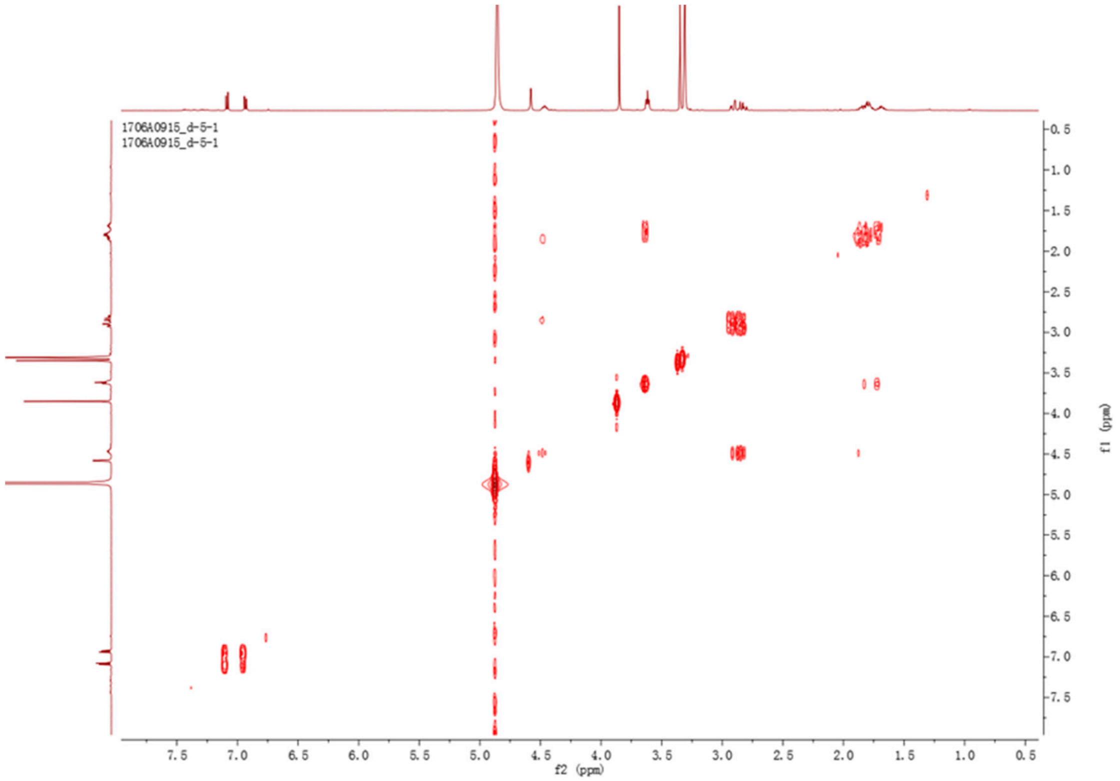

Fig.S10. ^{1}H-^{1}H COSY spectrum (CD_3OD, 600MHz) of compound (2)

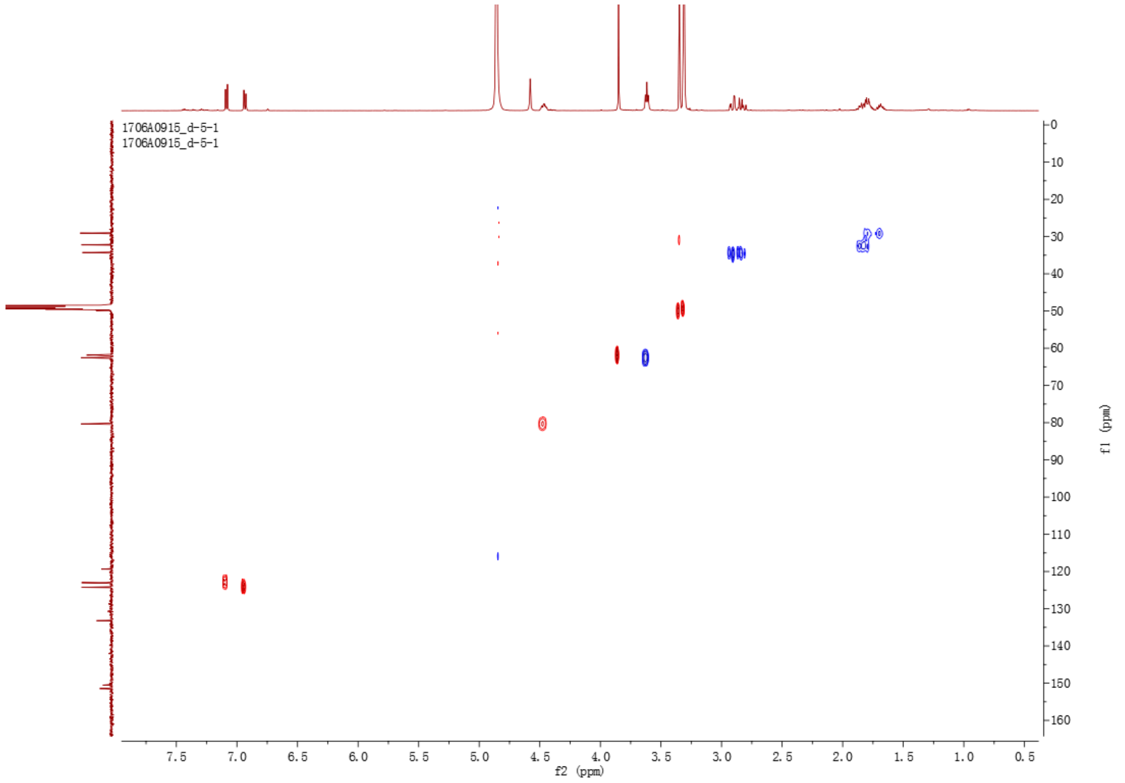

Fig.S11. HSQC spectrum (CD_3OD, 600MHz) of compound (**2**)

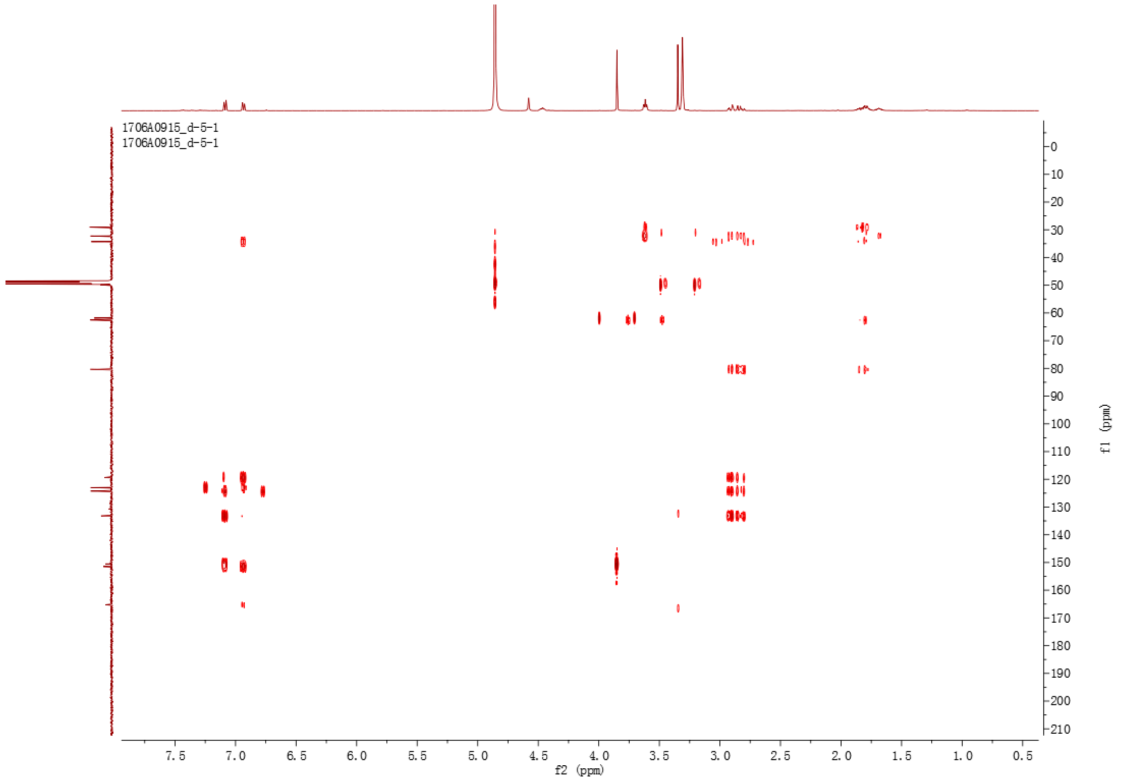

Fig.S12. HMBC spectrum (CD_3OD, 600MHz) of compound (**2**)

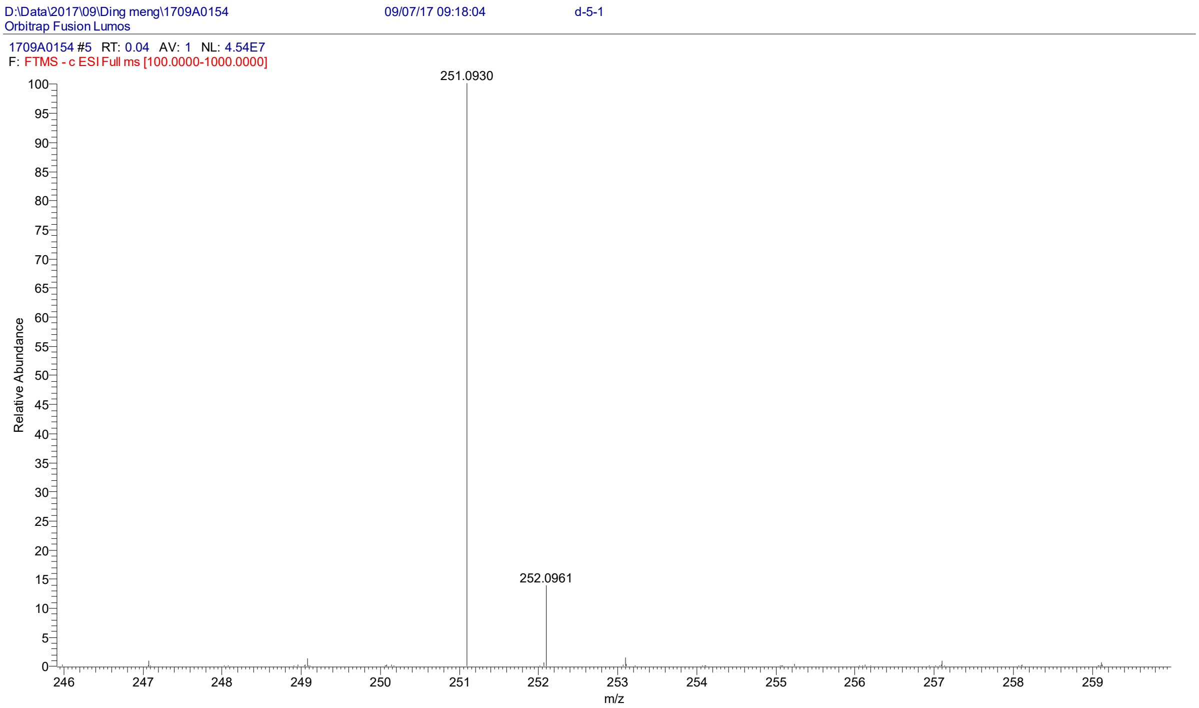

Fig.S13. HR-ESI-MS spectrum of compound (**2**)

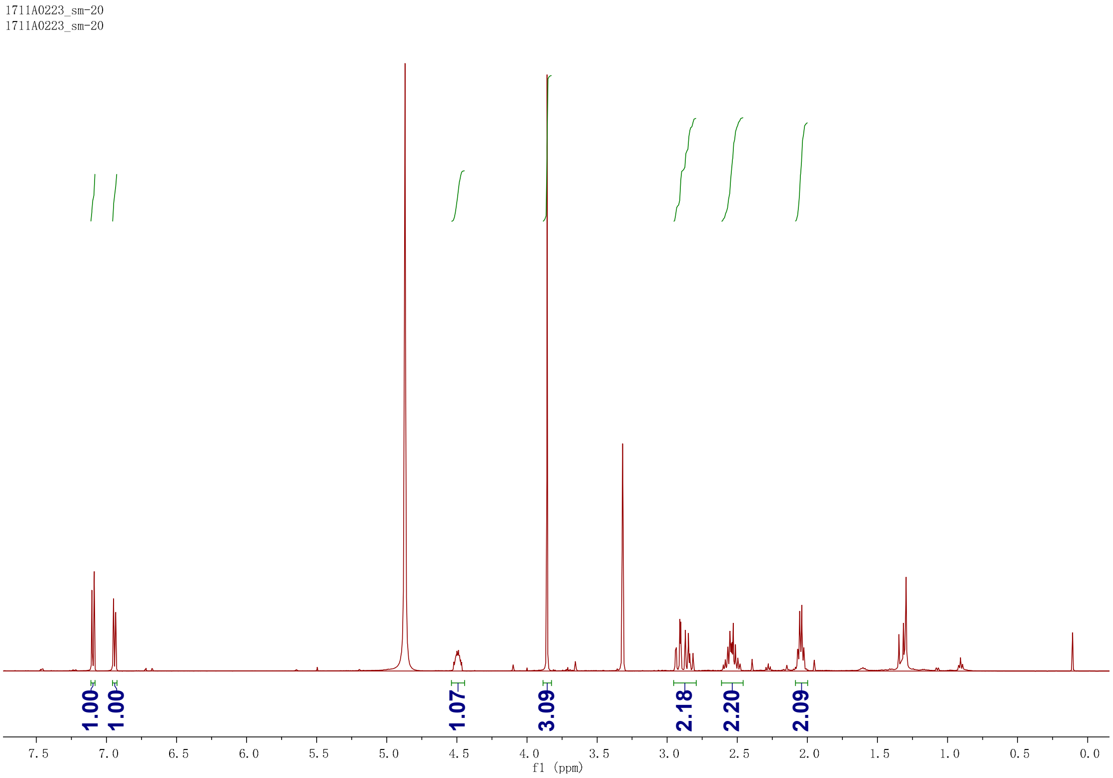

Fig.S14. ^{1}H- NMR spectrum (CD_3OD, 600MHz) of compound (**3**)

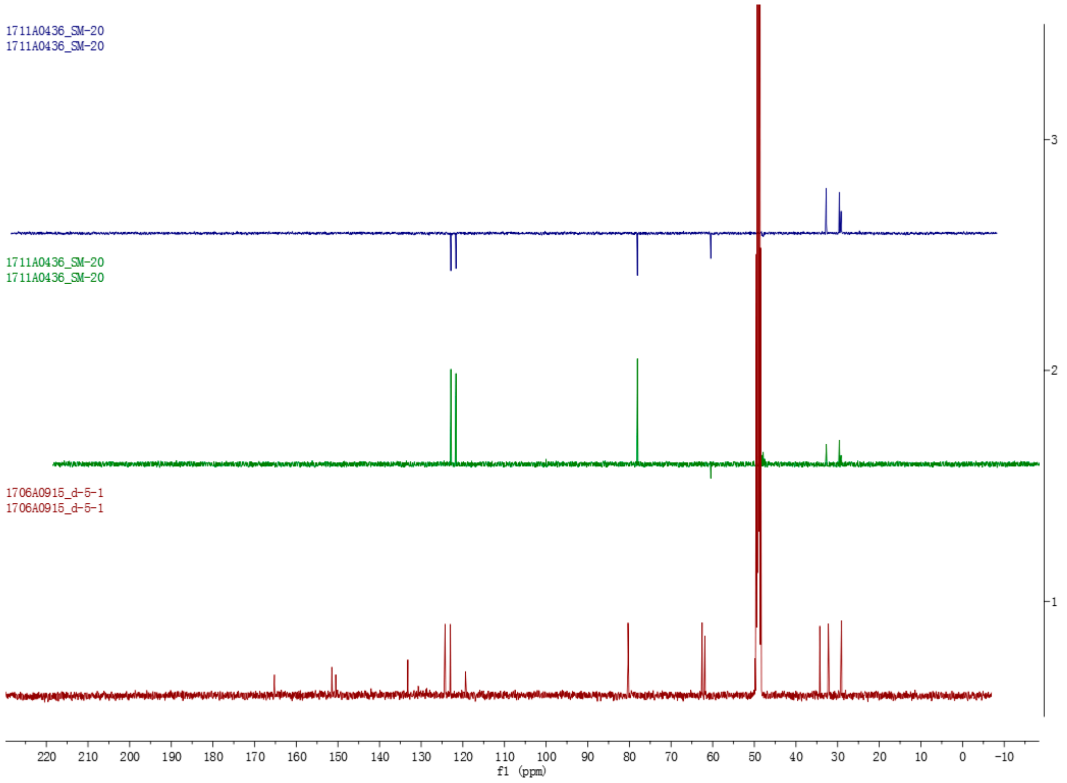

Fig.S15. DEPT 135, DEPT 90 and ^{13}C-NMR spectrum (CD_3OD, 150MHz) of compound (**3**)

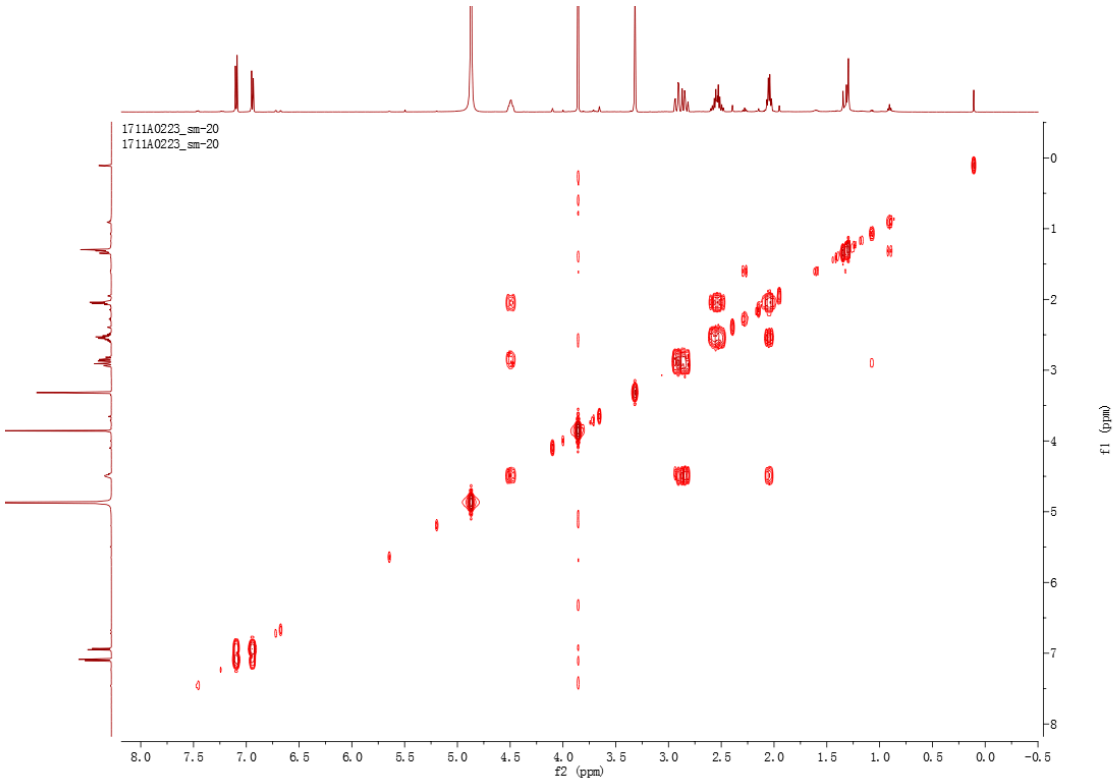

Fig.S16. ^{1}H-^{1}H COSY spectrum (CD_3OD, 600MHz) of compound (**3**)

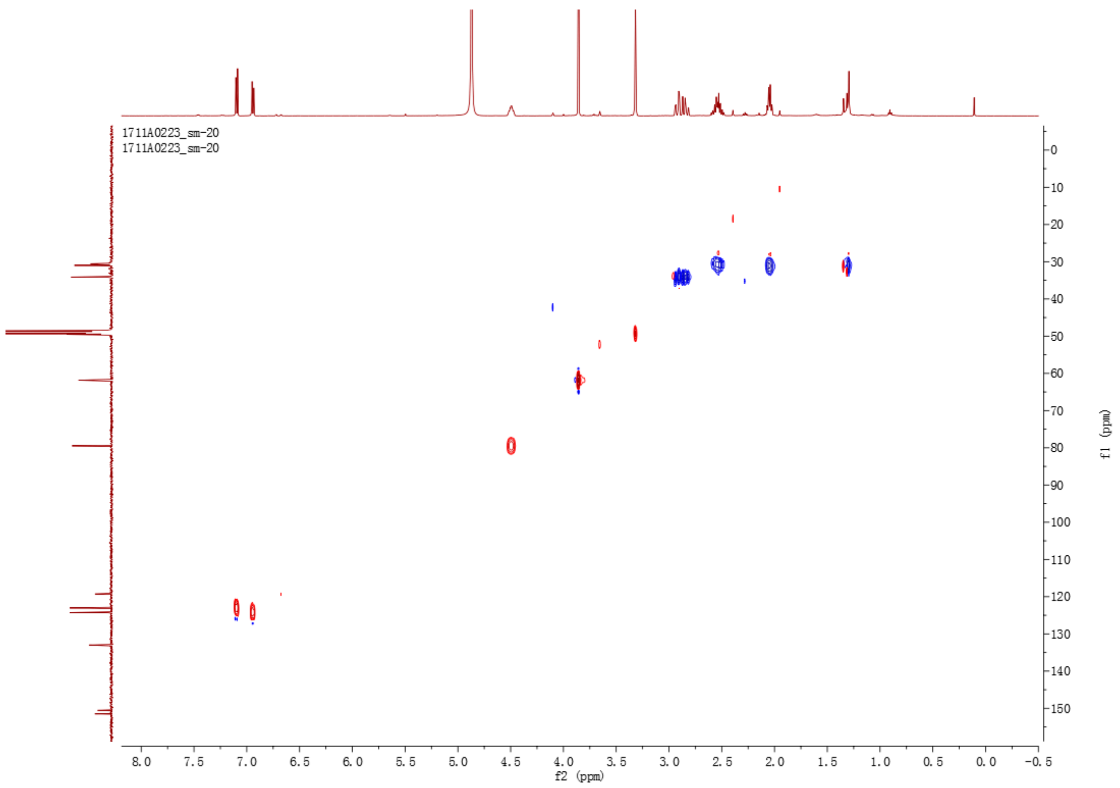

Fig.S17. HSQC spectrum (CD3OD, 600MHz) of compound (**3**)

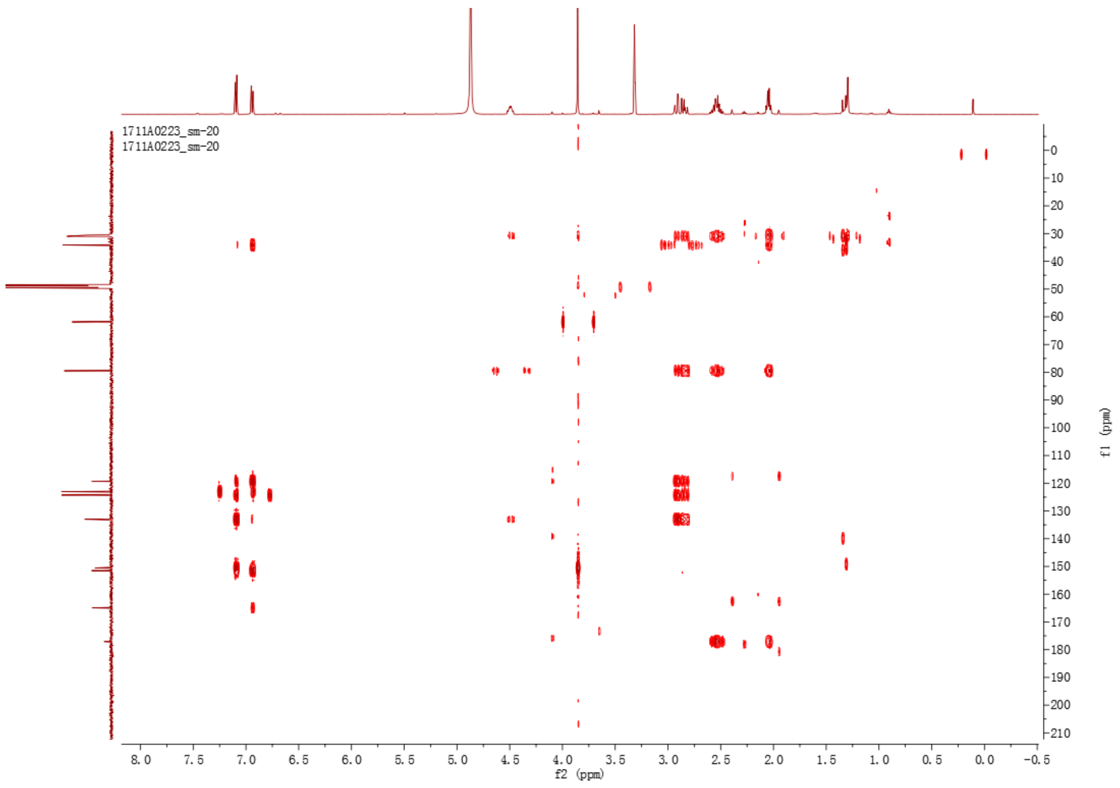

Fig.S18. HMBC spectrum (CD3OD, 600MHz) of compound (**3**)

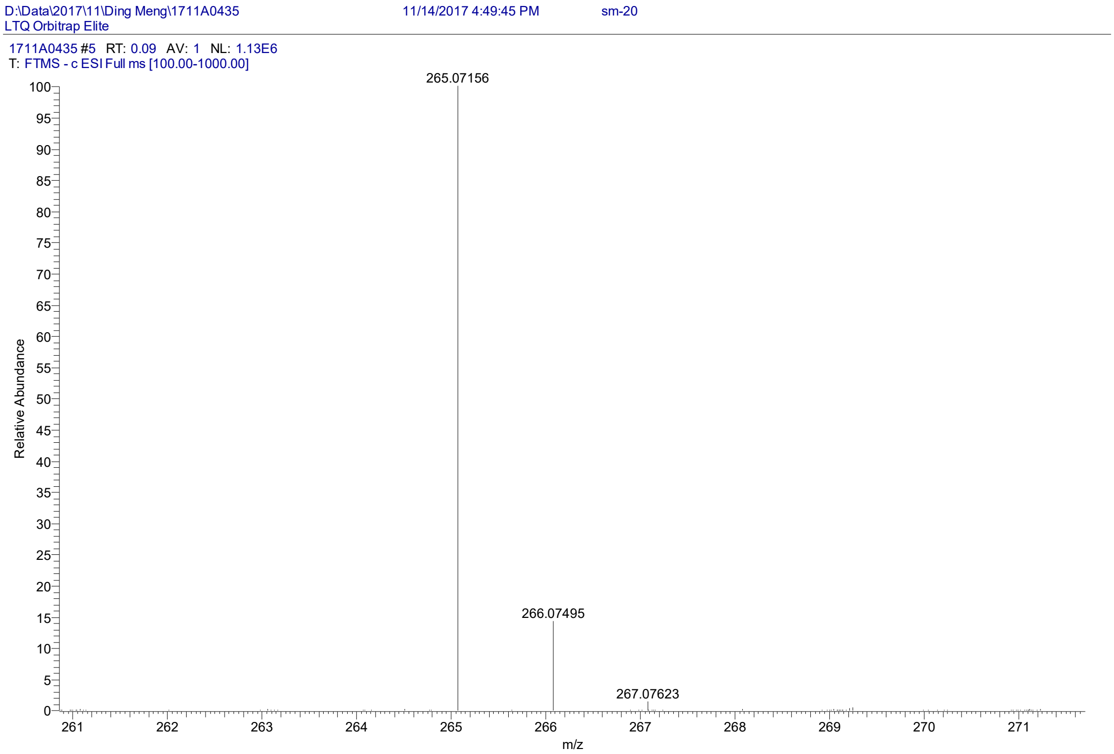

Fig.S19. HR-ESI-MS spectrum of compound (**3**)

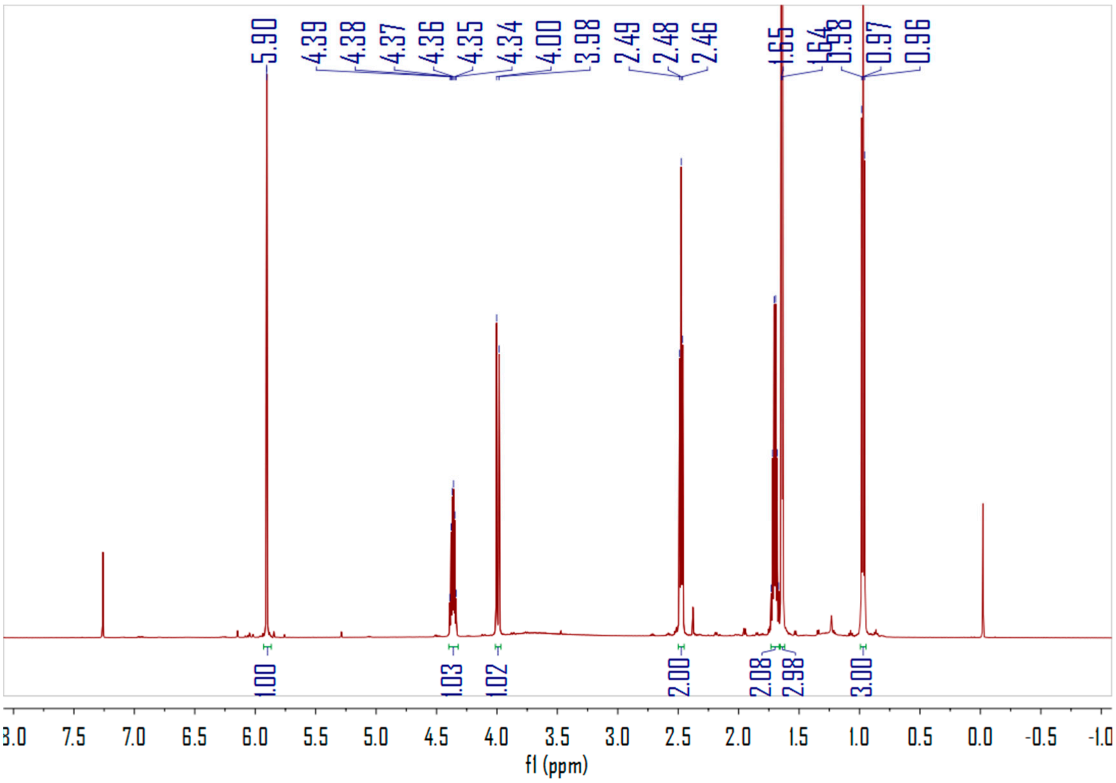

Fig.S20. ^{1}H- NMR spectrum ($CDCl_3$, 600MHz) of compound (**4**)

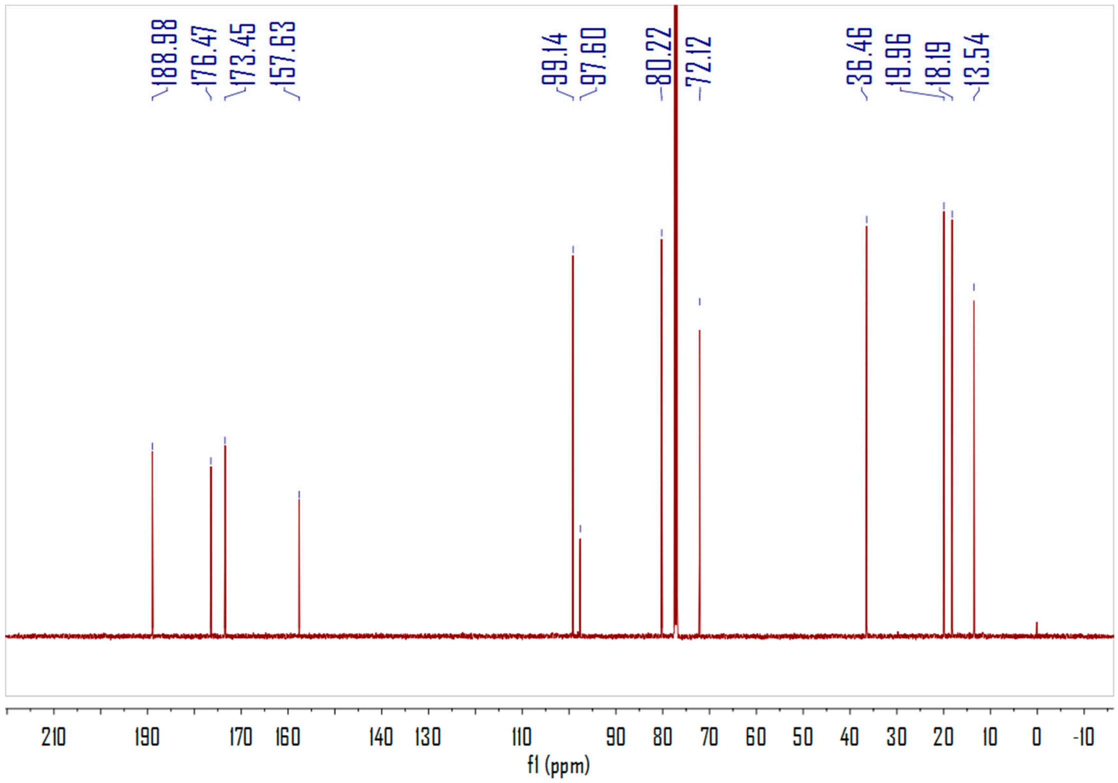

Fig.S21. ^{13}C- NMR spectrum ($CDCl_3$, 150MHz) of compound (**4**)

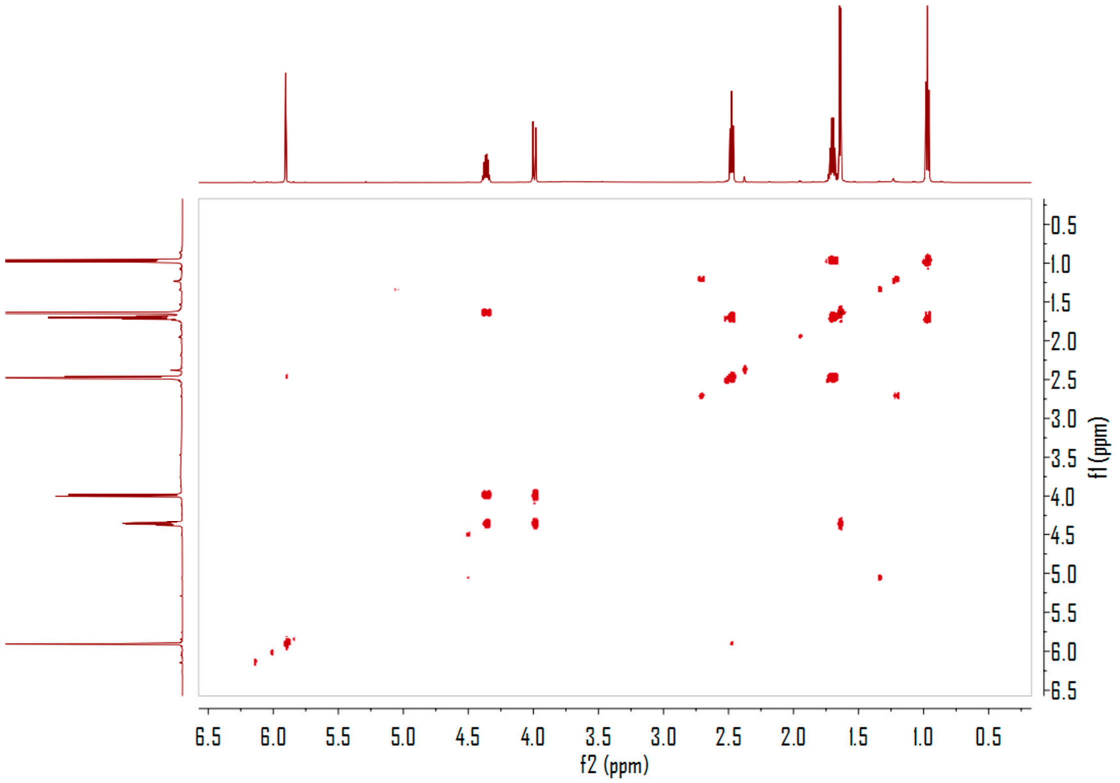

Fig.S22. ^{1}H-^{1}H COSY spectrum ($CDCl_3$, 600MHz) of compound (**4**)

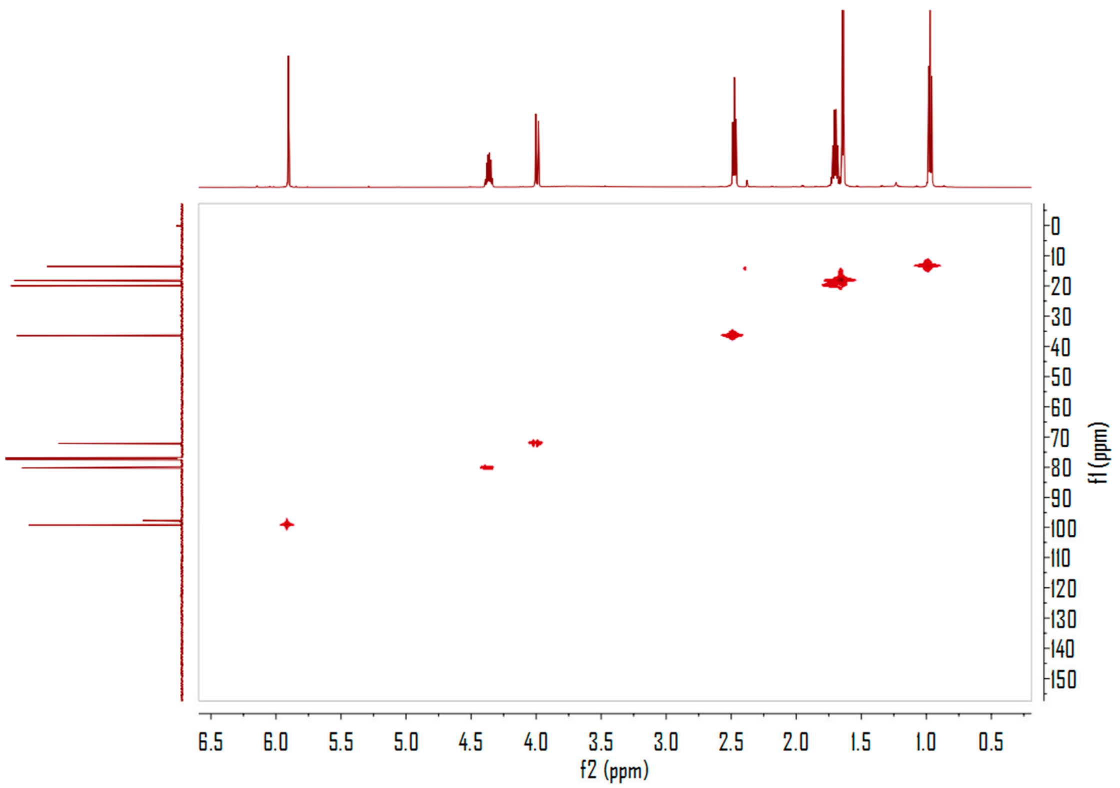

Fig.S23. HSQC spectrum ($CDCl_3$, 600MHz) of compound (**4**)

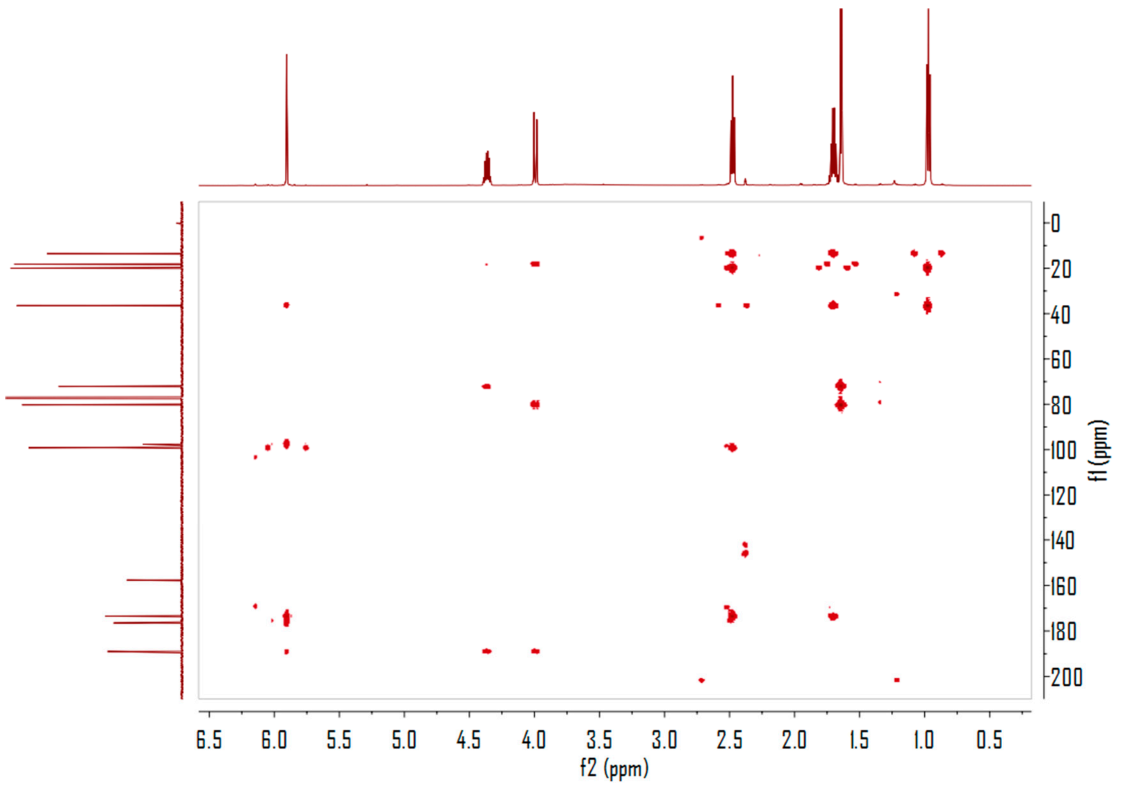

Fig.S24. HMBC spectrum ($CDCl_3$, 600MHz) of compound (**4**)

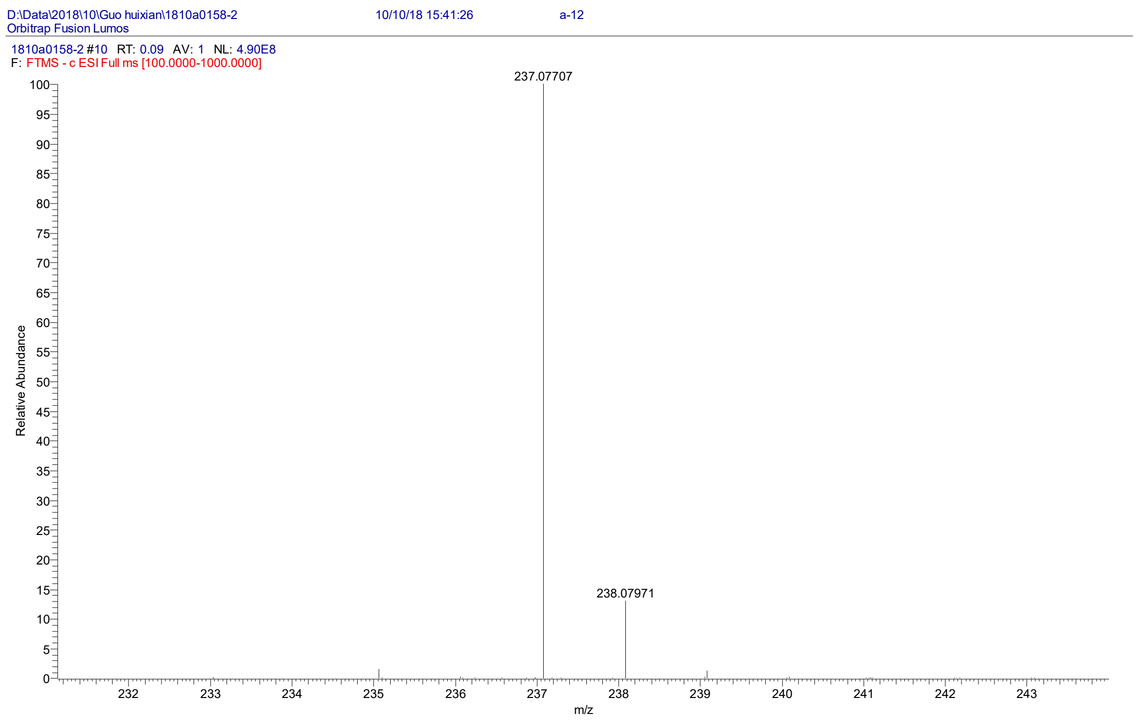

Fig.S25. HR-ESI-MS spectrum of compound (**4**)

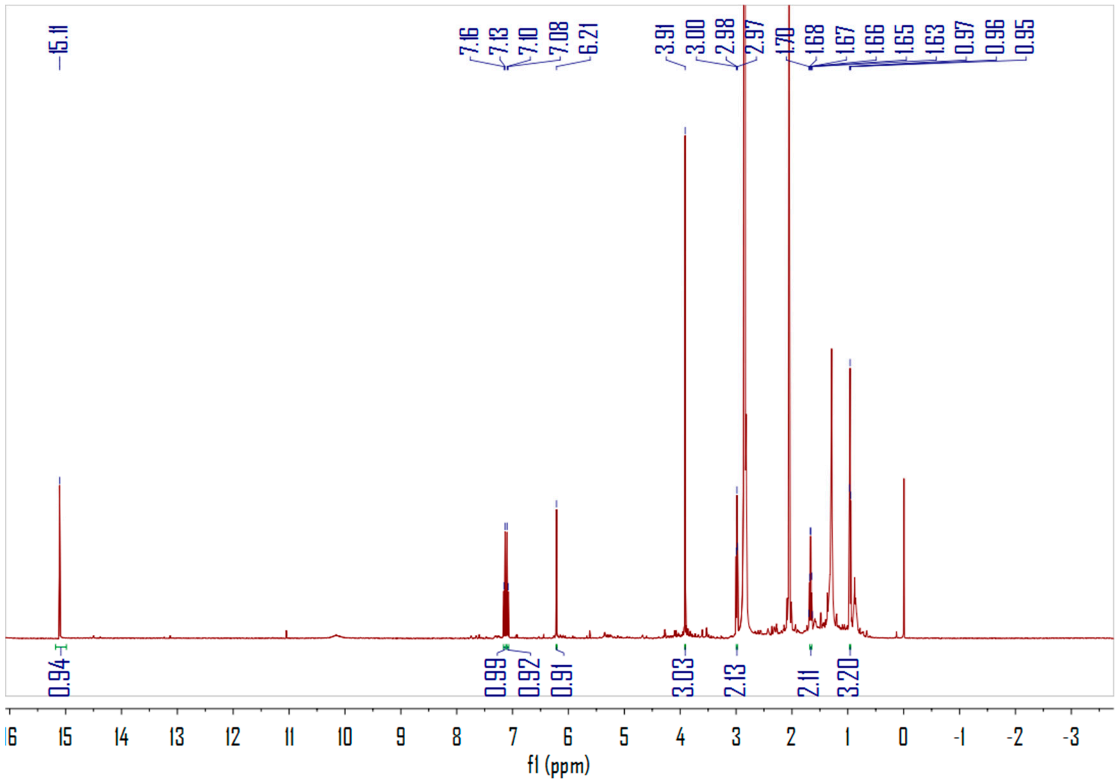

Fig.S26. ^{1}H- NMR spectrum (acetone-d_6, 600MHz) of compound (**7**)

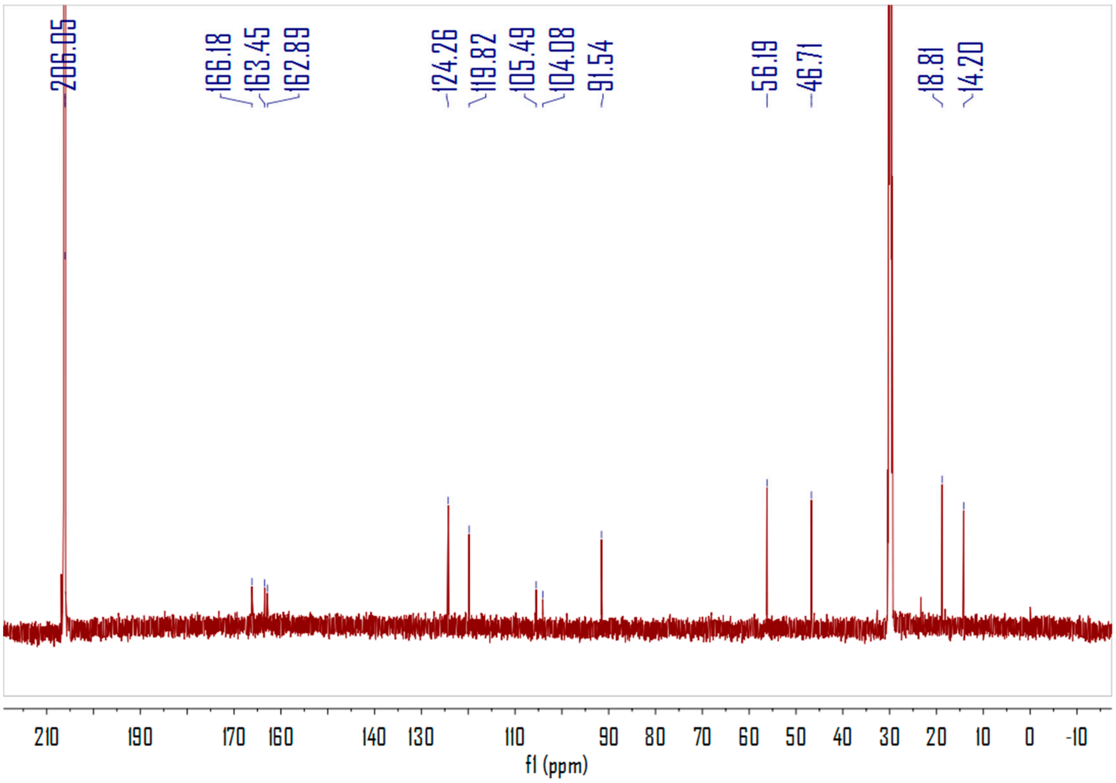

Fig.S27. ^{13}C- NMR spectrum (acetone-d_6, 150MHz) of compound (**7**)

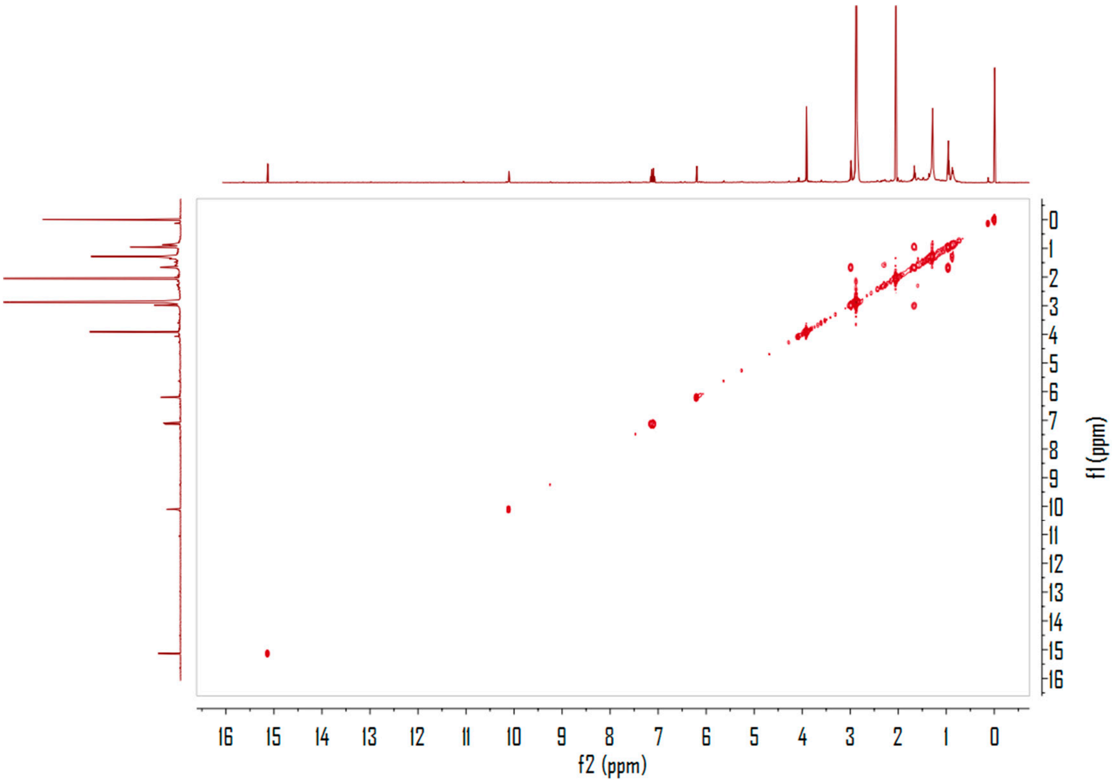

Fig.S28. ^{1}H-^{1}H COSY spectrum (acetone-d_6, 600MHz) of compound (**7**)

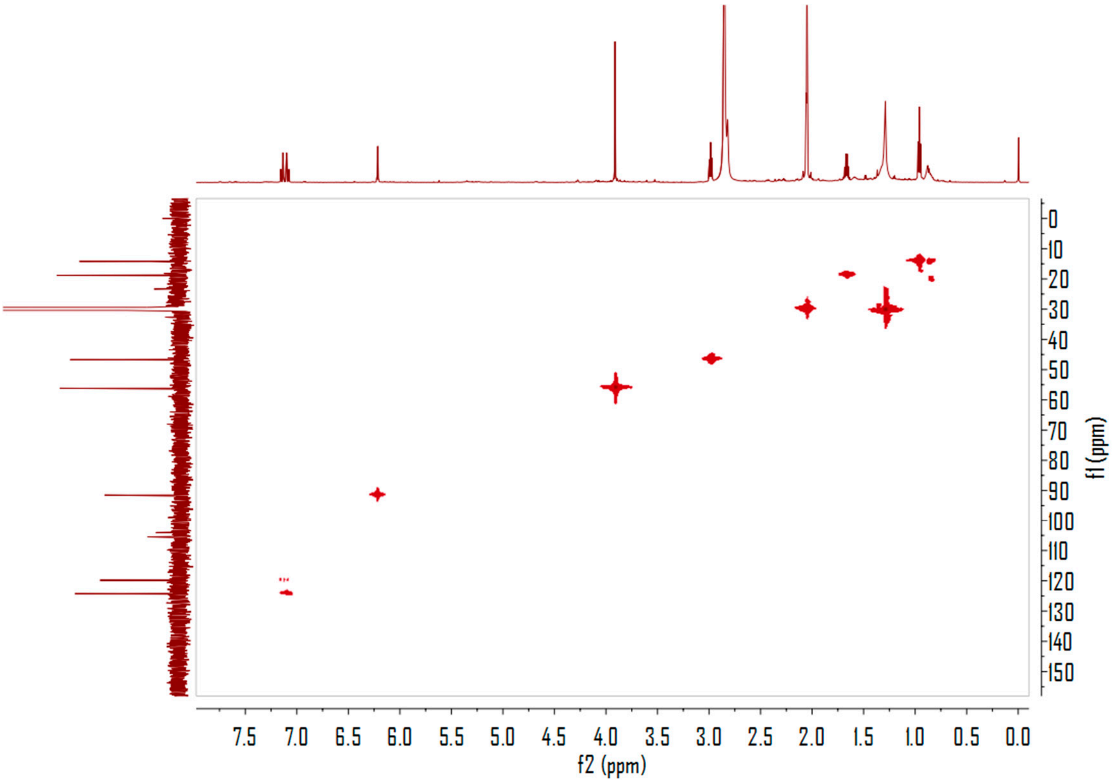

Fig.S29. HSQC spectrum (acetone-d_6, 600MHz) of compound (**7**)

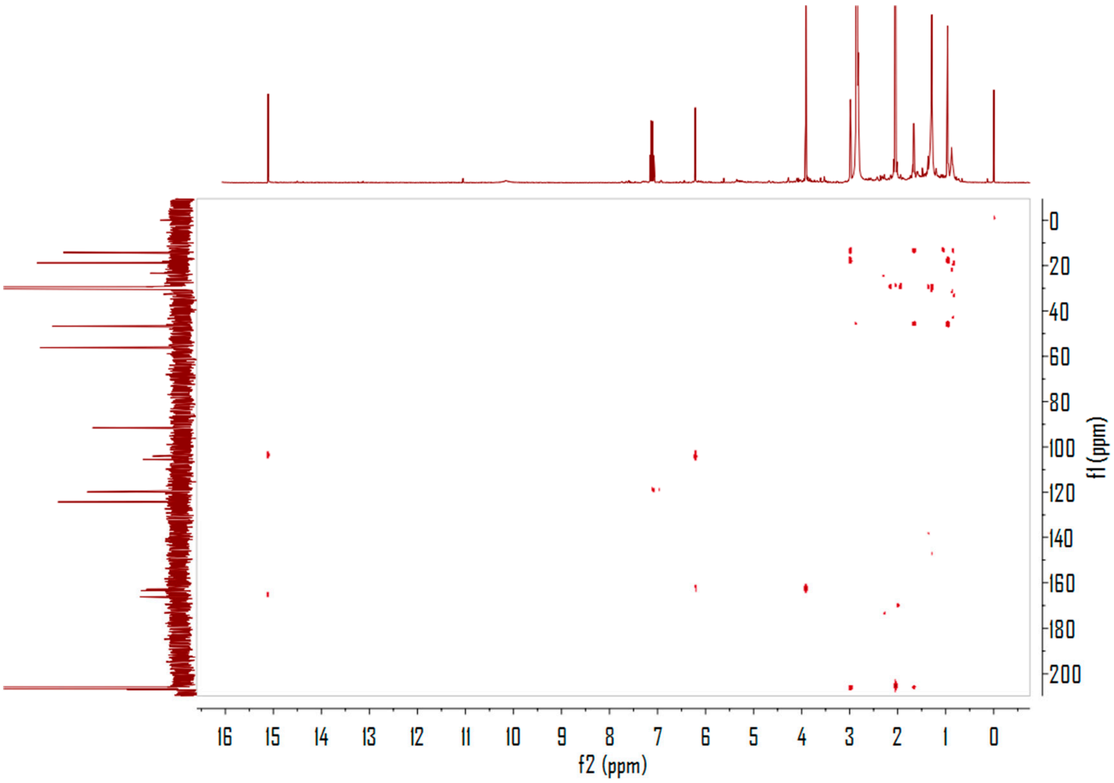

Fig.S30. HMBC spectrum (acetone-d_6, 600MHz) of compound (**7**)

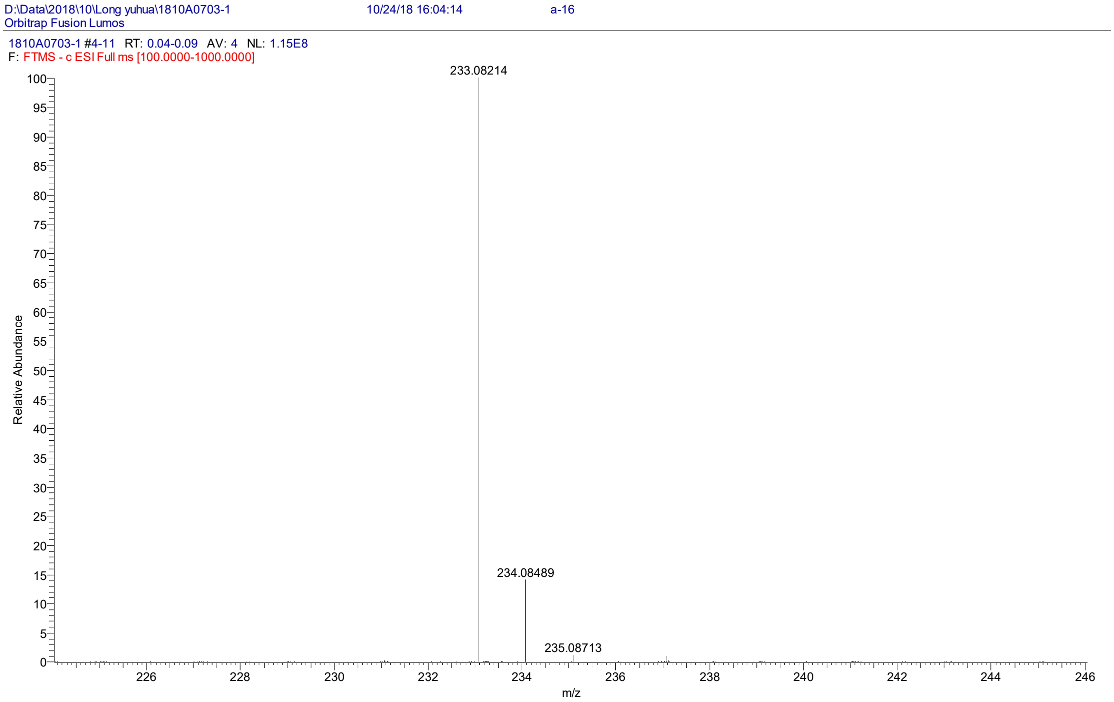

Fig.S31. HR-ESI-MS spectrum of compound (**7**)

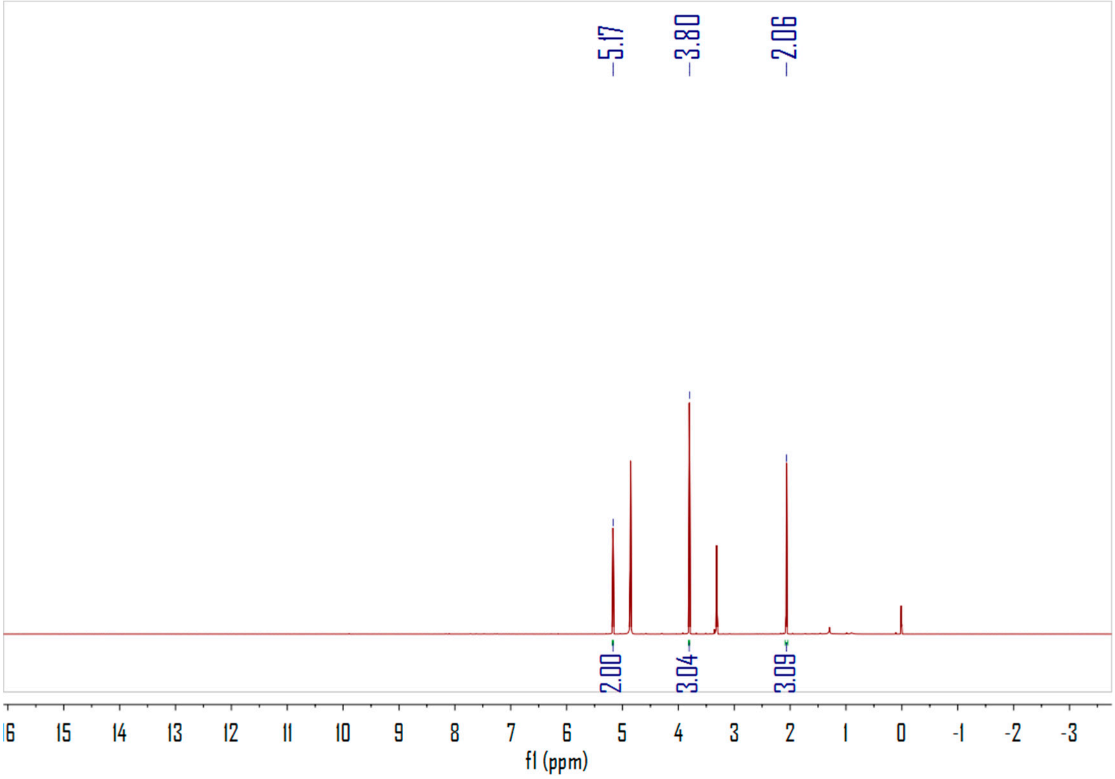

Fig.S32. ^{1}H- NMR spectrum (CD_3OD, 600MHz) of compound (**8**)

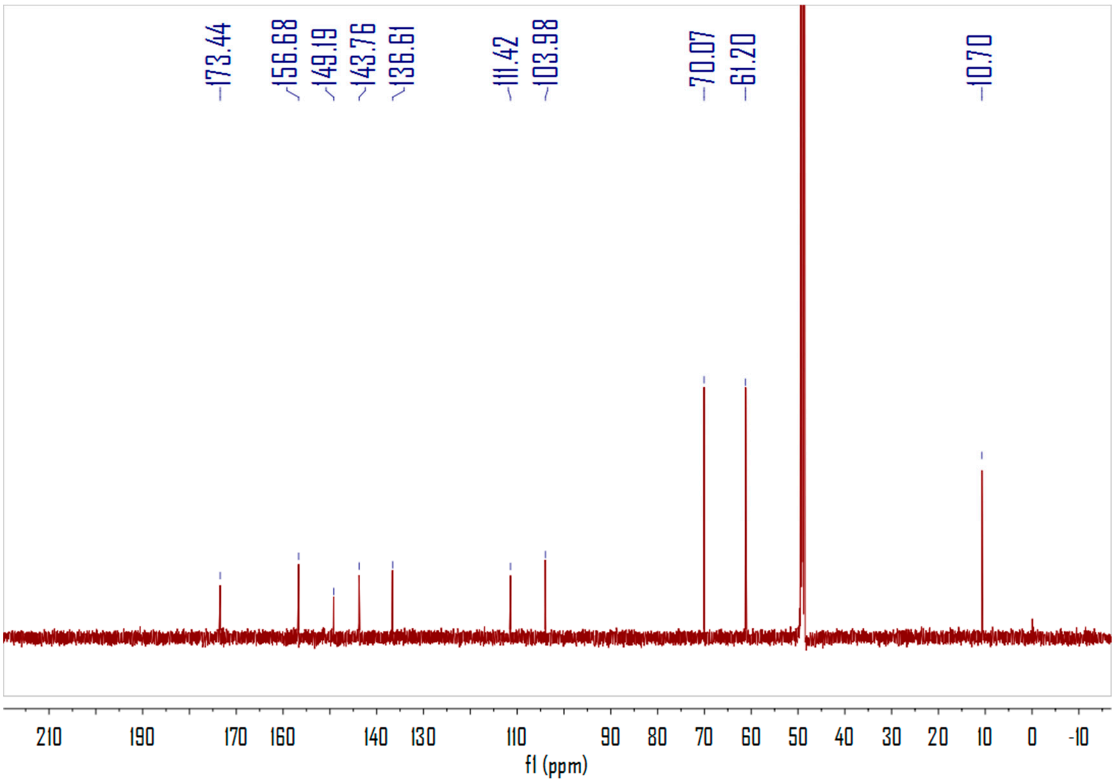

Fig.S33. ^{13}C- NMR spectrum (CD_3OD, 150MHz) of compound (**8**)

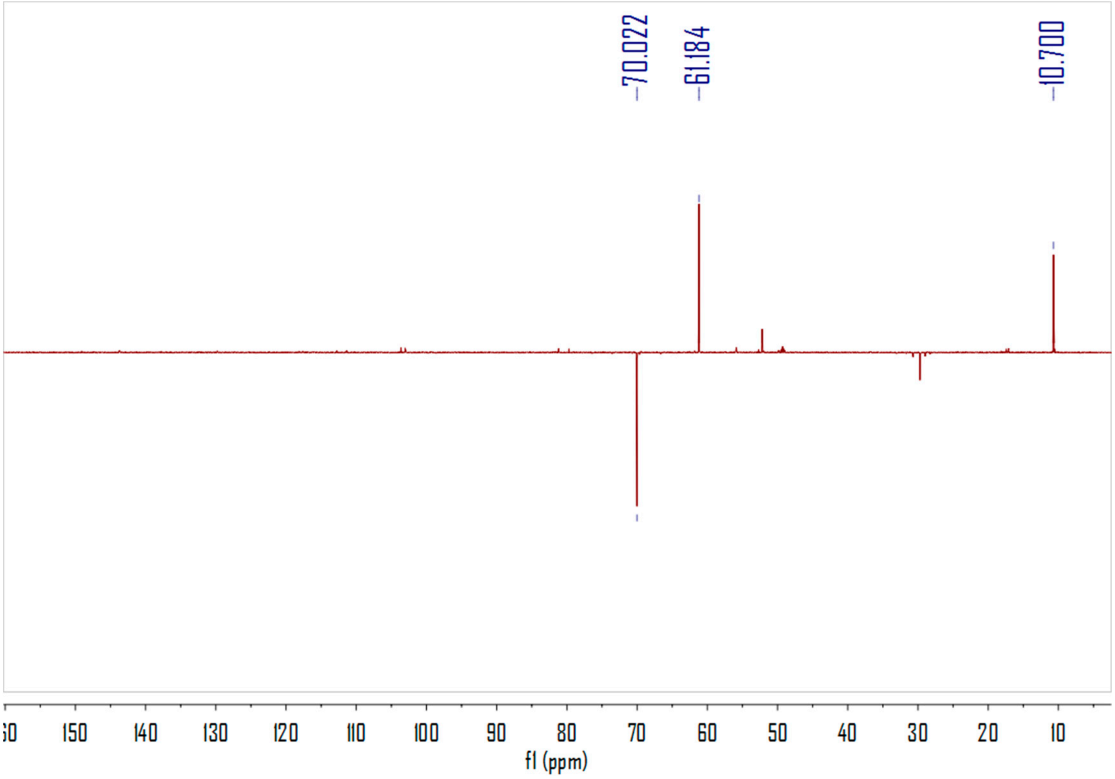

Fig.S34. DEPT 135 spectrum (CD_3OD, 150MHz) of compound (**8**)

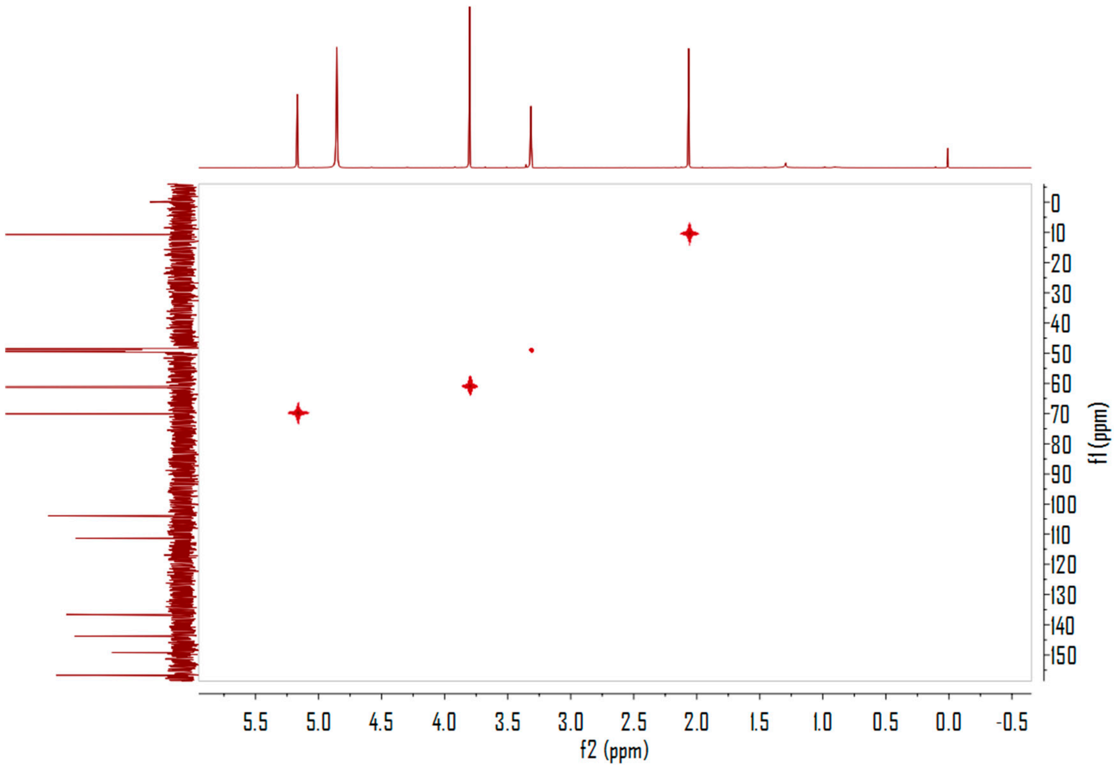

Fig.S35. HSQC spectrum (600MHz, CD_3OD) of compound (**8**)

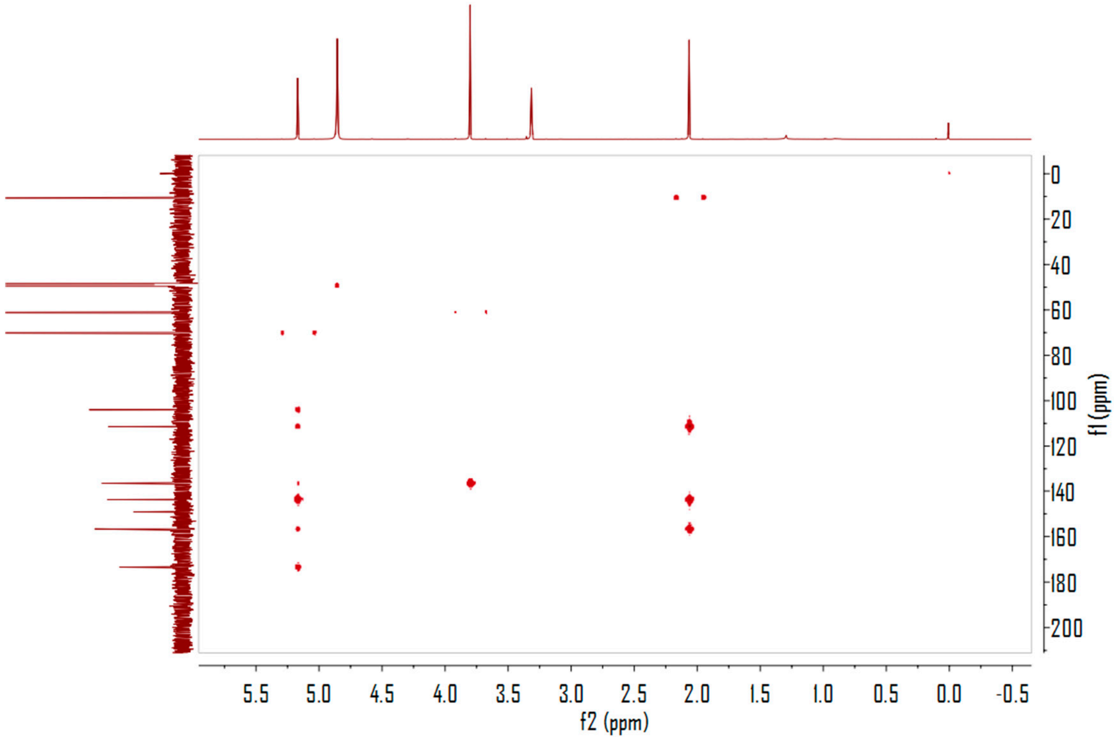

Fig.S36. HMBC spectrum (600MHz, CD_3OD) of compound (**8**)

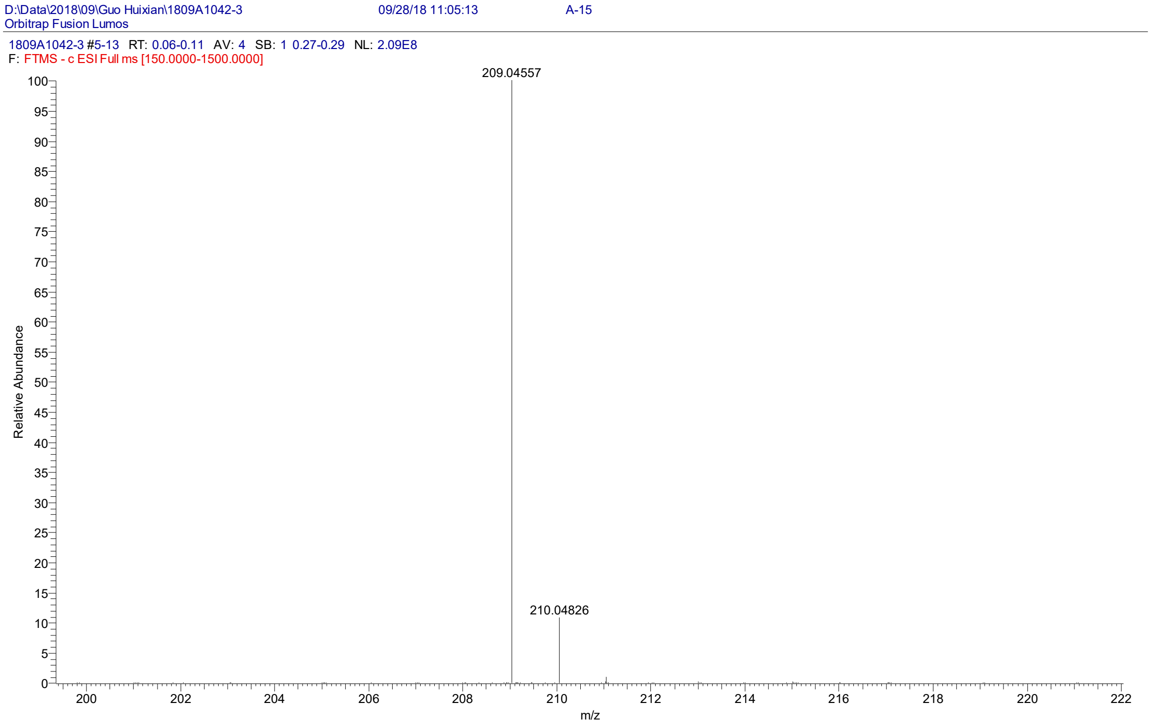

Fig.S37. HR-ESI-MS spectrum of compound (**8**)
